# Mechanistic Insight into Serine Flux Regulation through Nanoscale Organization of Glucose and Serine Transporters by Substrate Probe-Based Direct Stochastic Optical Reconstruction Microscopy Imaging

**DOI:** 10.34133/research.0805

**Published:** 2025-08-05

**Authors:** Pengwei Jiang, Hao Hou, Jiaqi Wang, Xumin Wang, Yaqi Wang, Simin Liu, Junling Chen, Hongda Wang, Feng Liang

**Affiliations:** ^1^ The State Key Laboratory of Refractories and Metallurgy, School of Chemistry & Chemical Engineering, Wuhan University of Science and Technology, Wuhan, Hubei 430081, P. R. China.; ^2^ Research Center of Biomembranomics, Key Laboratory of Electroanalytical Chemistry, Changchun Institute of Applied Chemistry, Chinese Academy of Sciences, Changchun, Jilin 130022, P. R. China.

## Abstract

Serine serves as a metabolic nexus in tumors, coordinating one-carbon metabolism, nucleotide synthesis, and redox regulation. While serine transporters (SerTs) are known to be dysregulated in cancer, their functional nanoscale organization remains unresolved due to the limitation of resolution imaging and available probes. Here, we developed a substrate-based fluorescent probe (Ser-probe) enabling direct stochastic optical reconstruction microscopy of SerTs, revealing malignancy-associated clustering assembly of SerTs that correlates with transport capacity. Compared to MDA-MB-231 cells, MCF7 cells with higher endogenous serine biosynthetic capacity exhibited more pronounced SerT/glucose transporter (GluT) co-clustering, suggesting that their spatial assemblies closely correlate with serine transport and biosynthetic functions in maintaining serine homeostasis. Their cluster morphology and co-assembly were revealed to depend critically on lipid rafts and glycan cross-linking, identifying the key determinants of spatial distribution to enable mechanistic understanding and potential regulation. Glucose deprivation weakened SerT/GluT clustering and their colocalization, which may be caused by their attenuated functional cooperativity in serine homeostasis maintenance under glucose-dependent suppression of serine synthesis. Pharmacological inhibition of phosphoglycerate dehydrogenase (PHGDH) initially enhanced SerT/GluT aggregation and colocalization, but this effect gradually attenuated as doses increased. The strategic combination of a PHGDH inhibitor with glucose restriction or free sialic acid synergistically disrupted SerT/GluT nanoscale organization, amplifying the anti-tumor efficacy of the PHGDH inhibitor and establishing the metabolic plasticity of transporter assemblies as a targetable vulnerability. This work establishes a fundamental link between transporter spatial assembly and tumor serine metabolic reprogramming, providing a new perspective to better understand SerT dysfunction in tumor metabolic reprogramming, offering novel therapeutic avenues for targeting serine metabolism in cancer.

## Introduction

Metabolic reprogramming is a hallmark of cancer progression, with serine metabolism emerging as a critical node that orchestrates multiple oncogenic processes [1,2]. As a versatile metabolic substrate, serine fuels nucleotide biosynthesis through one-carbon metabolism, maintains redox balance via glutathione production, and modulates epigenetic regulation through *S*-adenosylmethionine generation [3]. Targeting the vital regulators of serine metabolism represents a promising therapeutic strategy to exploit cancer-specific metabolic vulnerabilities, enabling both diagnostic detection and precision intervention in tumors with dysregulated serine pathways [4]. For example, the pharmacological inhibition of phosphoglycerate dehydrogenase (PHGDH), as the rate-limiting enzyme in de novo serine synthesis, has emerged as an attractive therapeutic target in recent years [5].

As a nonessential amino acid, cellular serine pools are dynamically maintained through the integration of both intracellular biosynthesis and extracellular transport via specialized serine transporters (SerTs) [6,7]. This dual-source regulation creates a metabolic flexibility that tumors exploit to evade single-pathway inhibition [4]. An important unanswered question is how cancer cells achieve coordination of these complementary pathways, especially through the functional interplay between spatial organization and metabolic transport systems.

Membrane biomolecules exhibit precisely organized spatial patterns that dictate their functional states, including activation, ligand binding, catalytic activity, and transport efficiency [8,9]. While some members of the SerT family have been observed in fluorescence imaging, a systems-level understanding of all SerTs’ spatial regulation remains elusive, constrained by the lack of specific probes and the low resolution limitations of traditional imaging techniques [10,11].

Super-resolution imaging, particularly stochastic optical reconstruction microscopy (STORM), has revolutionized the visualization of membrane protein organization at the nanoscale level, enabling unprecedented insights into molecular assembly mechanism and its relationship with molecular function [12–14]. A key determinant of STORM imaging fidelity lies in fluorescent probes [15]. Capitalizing on the high affinity and specificity of natural substrates for membrane transporters, substrate-based fluorescent probes have emerged as powerful chemical labeling tools for precise visualization of densely packed membrane proteins under minimal steric interference, compared with antibody probes [16].

Here, to systematically and accurately map SerT organization, we exploited the high affinity and specificity between serine and membrane SerTs to develop Ser-probe, a small-molecule fluorescent probe enabling direct stochastic optical reconstruction microscopy (dSTORM) imaging of all types of SerTs. This approach revealed cancer-stage-dependent nanoscale clustering of SerTs through comparative analysis of normal and progressively transformed malignant cells (MCF7 and MDA-MB-231 cells). Flow cytometric analysis of cellular serine uptake revealed a positive correlation between SerT clustering ability and transport capacity. Moreover, exogenous serine supplementation enhanced both cellular serine uptake and SerT clustering assembly, confirming that SerT spatial organization is closely related to its transport function. Given the differential PHGDH expressions between breast cancer cells MCF7 and MDA-MB-231 [17–20], we inferred that specific SerT assembly may be caused by distinct cellular dependencies on de novo serine synthesis versus transport-mediated uptake. We then performed dual-color dSTORM imaging of SerTs and glucose transporters (GluTs) on MCF7 and MDA-MB-231 cells by Ser-probe and Glu-probe labeling, which was successfully applied in the dSTORM imaging of GluTs [16]. PHGDH-high MCF7 cells (indicating active serine synthesis) exhibited enhanced GluT clustering and stronger colocalization of SerTs and GluTs; glucose restriction also reduced GluT clustering assembly and weaken the colocalization of SerTs and GluTs. This suggests that coordinated spatial organization of SerTs and GluTs reflects dual regulation of serine levels by biosynthesis and transport. To elucidate the mechanistic basis of SerT/GluT clustering, we assessed the role of lipid rafts as classical functional microdomains in SerT and GluT membrane assembly [21–23]. MβCD-mediated cholesterol depletion disrupted raft integrity [22], triggering disintegration of SerT/GluT clusters into dispersed monomers with residual nanodomains, as well as attenuated their colocalization. Given the glycosylation of both proteins [24–26], we next examined glycan-mediated stabilization. Enzymatic cleavage of *N*-glycans by peptide-*N*-glycosidase F disrupted glycoprotein cross-linking lattices [15], with dSTORM revealing markedly reduced clustering and colocalization. These data indicate that lipid rafts and glycan cross-linking synergistically stabilize supramolecular platforms that scaffold SerT/GluT cluster assembly and mutual localization. Considering that the spatial arrangement of 2 transporters is closely related to serine metabolism through serine synthesis and external absorption pathways, we further investigated the effect of drugs targeting serine synthesis on the spatial distribution of the 2 transporters. We found that PHGDH inhibition induced dose-dependent reorganization of SerT/GluT membrane microdomains, while low-dose inhibitor treatment enhanced both transporter clustering and colocalization; these effects exhibited gradual attenuation as the inhibitor concentration increased. We further explored whether it is possible to improve drug efficacy by regulating the spatial distribution of these 2 types of transporters. Strategic combination of a PHGDH inhibitor with glucose restriction or free sialic acid treatment synergistically disrupts SerT/GluT clustering organization, amplifying the anti-tumor efficacy of the PHGDH inhibitor and establishing the metabolic plasticity of transporter assemblies as a targetable vulnerability.

By employing Ser-probe-based dSTORM imaging, we not only identified SerT assembly patterns across breast cancer subtypes but also uncovered a coordinated regulatory mechanism between endogenous serine synthesis and exogenous uptake. In addition, systematic characterization of drug-induced SerT/GluT redistribution revealed stress-responsive membrane domain reorganization, providing mechanistic insights into serine flux rewiring during breast cancer progression.

## Results and Discussion

### Synthesis of a small-molecule fluorescent probe for super-resolution imaging of SerTs

**Fig. 1.**
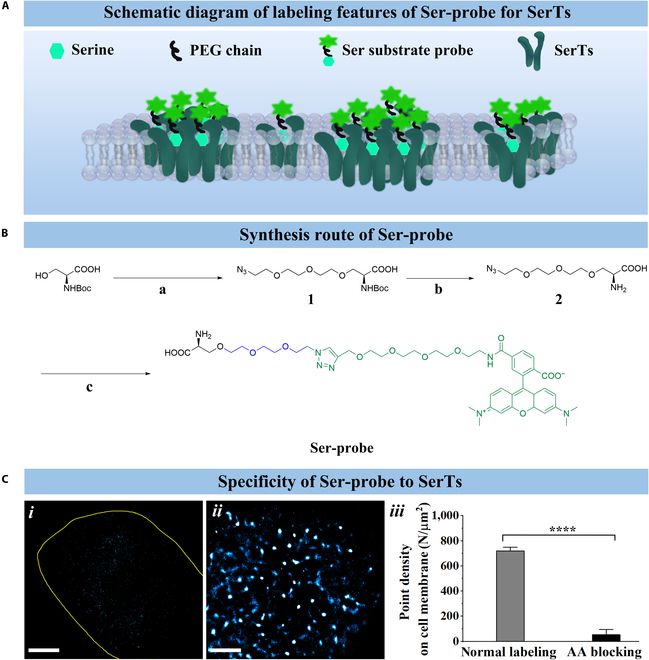
Synthesis and labeling properties of Ser-probe. (A) Labeling schematic representation of the serine probe to serine transporters (SerTs) on the cellular membrane. (B) Synthetic procedure of Ser-probe. Reagents and conditions: (a) 1-azido-2-(2-(2-iodoethoxy)ethoxy)ethane, NaH, dimethylformamide (DMF), room temperature (RT), 12 h; (b) dichloromethane (DCM)/trifluoroacetic acid (TFA) (v/v = 5:1), RT, 2 h; and (c) TAMRA-alkyne, CuSO_4_, tris(2-carboxyethyl)phosphine (TCEP), tris[(1-benzyl-1*H*-1,2,3-triazol-4-yl)methyl]amine (TBTA), phosphate-buffered saline (PBS), RT, 12 h, dark. (C) The direct stochastic optical reconstruction microscopy (dSTORM) imaging of SerTs on MCF10A membranes treated with free serine (*i*) or not (*ii*) with Ser-probe labeling and the corresponding histograms of average point density on the membrane (*iii*). Scale bars are 5 μm. All data were obtained from 10 cells in 3 independent experiments. Significant difference analyses were performed by using the unpaired dual-tailed *t* test, with “****” meaning *P* < 0.0001. PEG, polyethylene glycol; AA, amino acid.

To achieve high-resolution visualization of all kinds of SerTs on cell membranes, we engineered a small-molecule fluorescent probe by exploiting the native binding specificity and affinity of serine to its transporters. The probe was designed through strategic chemical modification of serine, preserving its critical transporter-binding motif while conjugating it to a photostable, high-performance fluorophore via a flexible linker (Fig. 1A). In detail, we selected (*tert*-butoxycarbonyl)-l-serine as a substrate of interest, linked the hydroxyl group of serine with an azide group through the polyethylene glycol chain as an outstanding flexible linker without affecting its specific binding ability as much as possible. After the deprotection reaction of the *t*-butoxycarbonyl group, it can be reacted with alkynyl TAMRA through the click chemistry reaction and finally the target small-molecule fluorescent probe, Ser-probe, was synthesized. The entire synthesis process of Ser-probe (Fig. 1B) was completed in 3 steps, and the details are described in the Supplementary Materials.

To validate the labeling specificity of Ser-probe, we conducted competitive binding assays with free serine using dSTORM imaging. The compared reconstructed dSTORM images (Fig. 1C, (*i*) and (*ii*)) virtually revealed that there was nearly no fluorescence signal on the treated membrane. The corresponding quantitative analysis of average point density on the cell membrane further confirmed a significant reduction in fluorescence signal on treated membranes (54 N/μm^2^) under successful competitive inhibition, compared with 721 N/μm^2^ on untreated MCF10A cells. This complete competitive inhibition confirmed the high binding specificity between Ser-probe and SerTs under physiological membrane conditions.

### Malignancy-specific clustering assembly of SerTs correlates with enhanced transport capacity

**Fig. 2.**
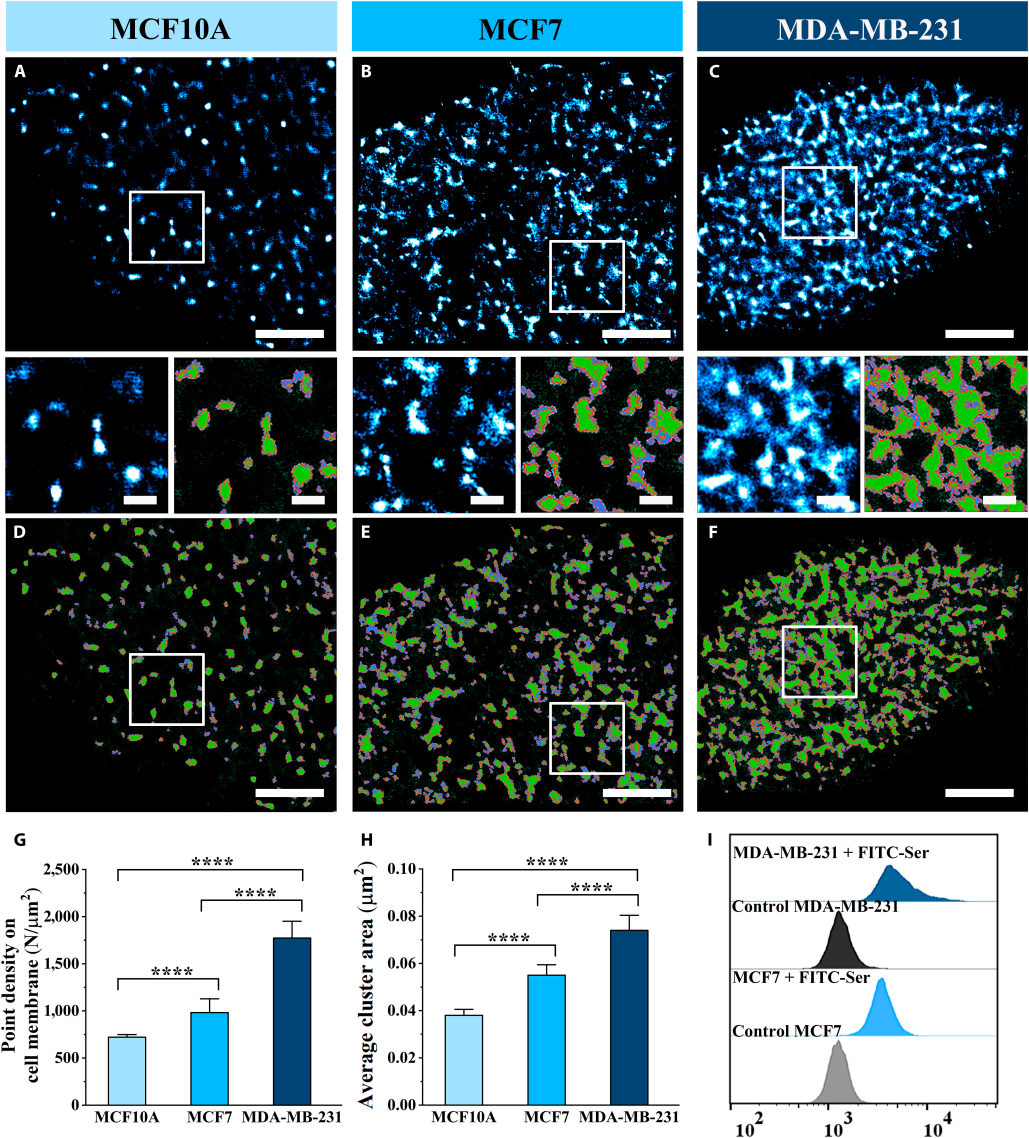
Compared distribution features of SerTs on normal and cancer cell membranes. (A to C) Compared dSTORM imaging of SerTs on MCF10A (A), MCF7 (B), and MDA-MB-231 (C) cells through Ser-probe labeling. (D to F) Corresponding maps of abstracted qualified clusters (in green) in SR-Tesseler analysis. (G and H) Compared histograms of the average point density on the cell membrane (G) and average cluster area (H). (I) Fluorescence analysis of fluorescein isothiocyanate (FITC)–Ser uptake by flow cytometry in MCF7 and MDA-MB-231 cells. All data were obtained from 10 cells in 3 independent experiments. Significant difference analyses were performed by using the unpaired 2-tailed *t* test, with “****” meaning *P* < 0.0001. Scale bars: 5 μm in original images and 500 nm in enlarged images.

To elucidate the malignancy-associated nanoscale reorganization of SerTs, we implemented dSTORM imaging to observe the fine distribution of SerTs on normal breast cells (MCF10A) and low/high-malignant breast cancer cells (MCF7/MDA-MB-231) with Ser-TAMRA labeling [27–30]. Firstly, we established 0.1 μM Ser-probe as the optimal labeling concentration for complete SerT labeling without off-target binding (Fig. S1). Then, super-resolution imaging revealed progressively enhanced SerT clustering during cancer progression (Fig. 2A to C). Quantitative analysis demonstrated significant increases in point density on the cell membrane across the cell lines: MCF10A showed sparse distribution (~721 N/μm^2^), while malignant MCF7 and highly metastatic MDA-MB-231 cells exhibited 1.4-fold (~982 N/μm^2^) and 2.5-fold (~1,773 N/μm^2^) increases, respectively (Fig. 2G).

To better quantify the detailed assembly patterns of SerTs, we further performed SR-Tesseler analysis [31] to acquire the qualified clusters (in green) extracted from the corresponding super-resolution images (Fig. 2D to F), which is able to precisely segment protein clusters by using a local density parameter and finally characterize protein organization at different scales. With SR-Tesseler analysis, we acquired the statistical data of average cluster area to characterize the degree of cluster size, showing ~0.038 μm^2^ on MCF10A, ~0.055 μm^2^ on MCF7, and ~0.074 μm^2^ on MDA-MB-231 (Fig. 2H); semiquantitative data of the average protein number in clusters were obtained by dividing the total fluorescent points by the mean probe relocalization frequency to assess SerT aggregation states, with ~4.03 N on MCF10A, ~7.24 N on MCF7, and ~11.42 N on MDA-MB-231 (Fig. S2A); the average coverage percentage of clusters, which assesses the clustering extent of SerT distribution on the cell membrane, also showed an increasing trend with 3.11% on MCF10A, 6.38% on MCF7, and 14.24% on MDA-MB-231 (Fig. S2B). All compared results indicate that as cells underwent malignant transformation, the clustering distribution of SerTs became more extensive, forming larger and more compact clusters. We speculate that the aggregation patterns of SerTs may serve as the primary functional platform, which can improve transport efficiency to fulfill the elevated serine metabolism in highly malignant cells. To test this hypothesis, we employed quantitative flow cytometry to assess the cellular uptake of Ser. Quantification of fluorescein isothiocyanate (FITC)–Ser uptake revealed enhanced accumulation in the mean fluorescence intensity (MFI) of MCF7 cells (4,653) versus controls (964) (Fig. 2I), while MDA-MB-231 cells exhibited significantly greater uptake, with MFI increasing from 1,039 to 7,456, establishing superior serine transport efficiency in MDA-MB-231 cells. These results collectively support a positive correlation between the SerT clustering assembly pattern and its cellular transport efficiency.

To further validate this relationship, we increased extracellular Ser concentrations and assessed corresponding changes in both the transport capacity and assembly patterns of SerTs (Fig. S3) [32,33]. Quantitative flow cytometry confirmed that elevating extracellular Ser enhanced cellular uptake, as evidenced by increased MFI (from 4,653 to 6,077) (Fig. S4). Concurrently, comparative dSTORM imaging revealed significantly enhanced clustering of SerTs on serine-supplemented cells (Fig. S3A and B). The detailed statistical results showed that the average point density on the membrane of SerTs increased from ~982 to ~1,401 N/μm^2^ (Fig. S3C), the average cluster area increased from ~0.055 to ~0.076 μm^2^ (Fig. S3D), the average molecule number per cluster was also enhanced from ~7.24 to ~10.89 N (Fig. S3E), and the average cluster coverage percentage enlarged from ~6.38% to ~11.72% (Fig. S3F). Increased serine uptake and enhanced cluster assembly under high-concentration-serine stimulation further support the proportional relationship between SerT assembly pattern and transport capacity. Therefore, the specific assembly of SerTs, closely related to their functions, could act as potential indicators for assessing the occurrence and progression of cancer.

### SerT/GluT clustering cooperativity sustains cellular serine homeostasis

**Fig. 3.**
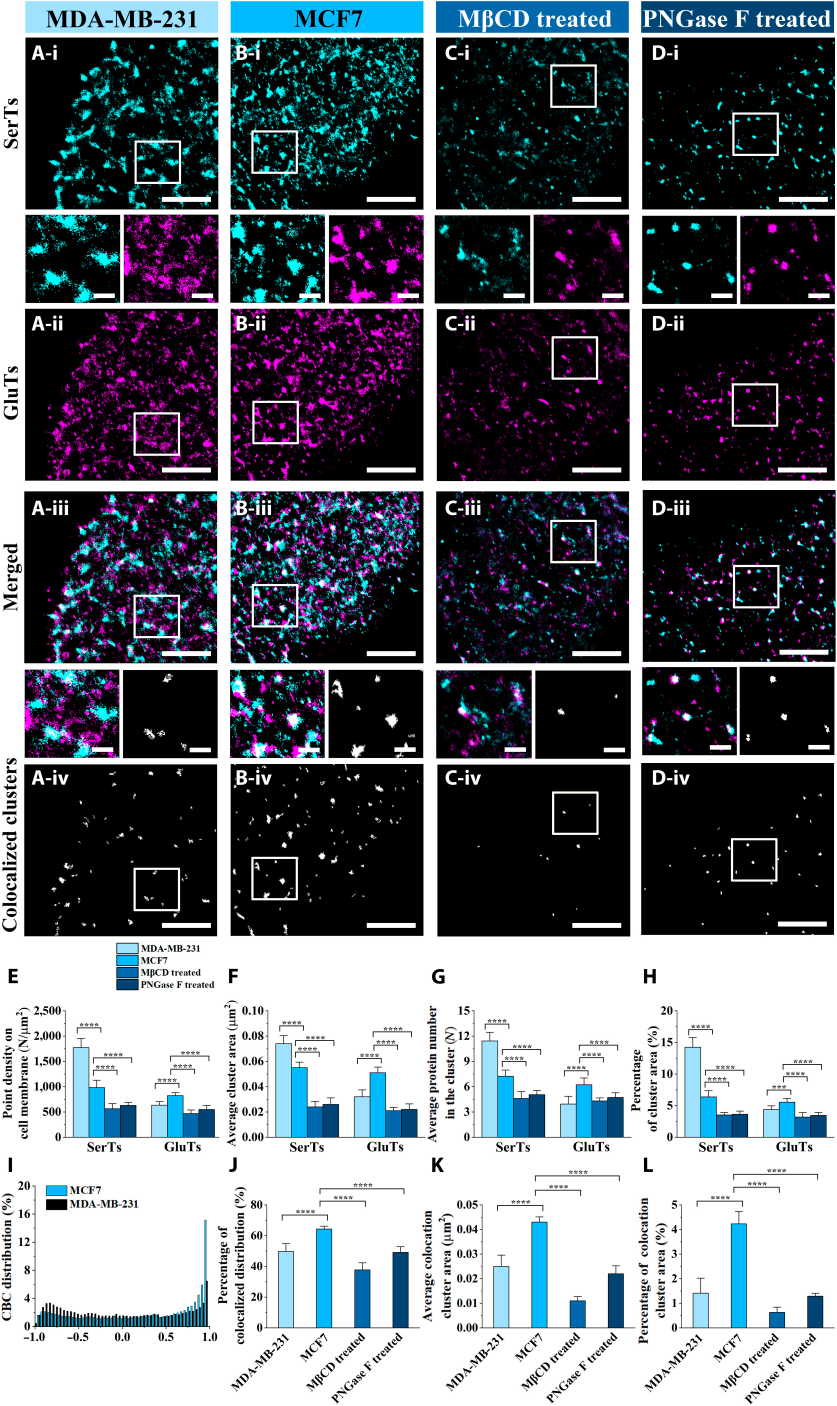
Dual-color dSTORM imaging of SerT/glucose transporter (GluT) distributions across cell types and treatments. (A to D) Single-channel dSTORM reconstruction images (*i* and *ii*) and merged images (*iii*) of SerT (cyan) and GluT (magenta) distributions on MDA-MB-231 cells (A), MCF7 cells (B), and MCF7 cells treated with MβCD (C) or peptide-*N*-glycosidase F (PNGase F) (D), as well as the corresponding maps of the colocalized regions (*iv*). (E to H) Compared histograms of the average point density on cell the membrane (E), average cluster area (F), average protein number in clusters (G), and average cluster coverage (H) on MDA-MB-231, MCF7, and MCF7 cells treated with MβCD and PNGase F. (I to L) Comparison of coordinate-based colocalization (CBC) value distributions (I), the proportion of *V*_CBC_ (0 < *V*_CBC_ ≤ 1) (J), average cluster area (K), and coverage percentage (L) of the colocalized regions. All data are the statistical results of more than 10 cells from 3 independent experiments. Significant difference analyses were performed by using the unpaired 2-tailed *t* test, with “***” meaning *P* < 0.001 and “****” meaning *P* < 0.0001. Scale bars are 5 μm in original images and are 500 nm in enlarged images.

In order to identify the regulators of SerT nanoscale organization, we leveraged differential PHGDH expression in MCF7 versus MDA-MB-231 cells (DepMap: 6.92 vs 2.29 [depmap.org]) as an indicator of serine synthesis divergence, testing its contribution to distinct SerT assemblies. Given glucose-derived serine biosynthesis coupled with external transportation to maintain cellular serine homeostasis [18], we quantitatively compared spatial patterning and colocalization of SerTs and GluTs by dual-color dSTORM imaging. The reconstructed dSTORM images from a single-color channel displayed that as the degree of cellular malignancy decreased (from MDA-MB-231 to MCF7), SerTs weakened the clustering distribution (Fig. 3A (*i*) and B (*i*)), while GluTs enhanced the clustering distribution (Fig. 3A (*ii*) and B (*ii*)). Quantitative analysis of GluT assembly further showed that compared with MCF7 cells, MDA-MB-231 cells exhibited a 22.96% reduction in average point density on the cell membrane, 37.25% shrinkage in cluster area, a 36.76% decline in intracluster protein density, and a 20.9% decreased spatial cluster coverage degree (Fig. 3E to H). Building on the assembly–transport functional linkage, we propose a compensatory regulation model: In MCF7 cells, enhanced GluT clustering facilitates efficient glucose uptake, supporting robust serine synthesis, while reduced SerT assembly weakens external transport. Conversely, MDA-MB-231 cells exhibit attenuated GluT clustering, which may constrain biosynthesis, but amplified SerT assembly can enhance external transport. This differential spatial organization enables both cell types to maintain serine homeostasis through complementary SerT/GluT transport tuning.

The merged dSTORM images of SerTs and GluTs display the clear detailed spatial relationship of these 2 types of transporters. We found a higher degree of colocalization (white part) of GluTs and SerTs on MCF7 (Fig. 3B (*iii*)) than on MDA-MB-231 (Fig. 3A (*iii*)), which was further displayed by enlarged images and the corresponding maps of colocalized clusters (Fig. 3A (*iv*) and B (*iv*)). Coordinate-based colocalization (CBC) analysis as a common and recognized analysis method was utilized to quantitatively characterize the spatial relationship between SerTs and GluTs, with the normalized CBC value (*V*_CBC_) ranging from −1 (anticorrelated) to 1 (perfectly correlated), with 0 indicating no spatial association [34]. CBC analysis revealed significantly higher colocalization (0 < *V*_CBC_ ≤ 1) between SerTs and GluTs in MCF7 cells (64.27%) versus MDA-MB-231 cells (49.70%) (Fig. 3I and J). Quantitative comparisons demonstrated larger cluster areas of colocalized region (~0.043 μm^2^ vs ~0.025 μm^2^) (Fig. 3K) and greater membrane coverage (~4.23% vs ~1.41%) (Fig. 3L) in MCF7 cells. These data indicate that MCF7 cells with the PHGDH-higher phenotype exhibits a more remarkable colocalization of SerTs and GluTs on membranes. We propose that this enhanced colocalization reflects coupled microdomains where glucose flux (via GluTs) and serine transport (via SerTs) synergistically maintain serine homeostasis. Elevated PHGDH expression in MCF7 cells likely enhances de novo serine biosynthesis, thereby tightening the functional coupling between glucose uptake and serine transport. This metabolic interdependence drives the observed co-clustering, forming spatially organized channels that optimize serine flux regulation.

As cholesterol-rich membrane microdomains that scaffold membrane protein assembly, lipid rafts represent critical functional domains for spatial organization [21–23]. To define their role in SerT/GluT assembly patterns, we disrupted rafts via MβCD-mediated cholesterol depletion [22]. This intervention triggered disintegration of SerT/GluT clusters, shifting organized assemblies to dispersed monomers and residual nanodomains (Fig. 3C (*i*) and (*ii*)). Quantification confirmed that the average point density on the membrane decreased by 42.67% (SerTs) and 43.26% (GluTs), the cluster area contracted by 56.36% (SerTs) and 58.82% (GluTs), the average molecule number in clusters shrunk by 36.33% (SerTs) and 30.81% (GluTs), and the average cluster coverage on the cell membrane declined by 44.83% (SerTs) and 42.52% (GluTs) (Fig. 3E to H). Concomitantly, the merged images displayed that raft disruption severely compromised SerT–GluT colocalization (Fig. 3C (*iii*) and (*iv*)), with *V*_CBC_ coefficients (0 < *V*_CBC_ ≤ 1) decreasing from 64.27% (normal cells) to 37.73% (Fig. 3J and Fig. S5A). Co-assembled domains exhibited marked contraction from 0.043 to 0.011 μm^2^ in area and from 4.23% to 0.63% in membrane coverage (Fig. 3K and L). These results definitively establish cholesterol-rich lipid rafts as biophysical scaffolds that stabilize SerT/GluT co-assemblies and mechanistically couple their spatial organization to transport regulation.

Given the glycosylated nature of SerTs and GluTs, we investigated glycan-mediated cross-linking in cluster stabilization [24–26]. Using peptide-*N*-glycosidase F to enzymatically cleave *N*-glycans, we disrupted glycoprotein lattice formation [15]. dSTORM imaging revealed significant dissolution of SerT/GluT clusters under posttreatment, with reduced membrane distribution and disassembled oligomeric states (Fig. 3DI (*i*) and (*ii*)). Quantitative analysis demonstrated that the average point density on the membrane decreased by 35.85% (SerTs) and 33.41% (GluTs), the cluster area contracted by 52.73% (SerTs) and 54.9% (GluTs), the average molecule number in clusters shrunk by 30.25% (SerTs) and 24.24% (GluTs), and the average cluster coverage on the cell membrane declined by 43.1% (SerTs) and 37.66% (GluTs) (Fig. 3E to H).

Additionally, the merged images from dual-color dSTORM imaging (Fig. 3D (*iii*) and (*iv*)) showed that SerT–GluT colocalization was substantially impaired: *V*_CBC_ coefficients decreased from 64.27% to 49.12% (Fig. 3J and Fig. S5B), while colocalized domain area contracted from ~0.043 to ~0.022 μm^2^ and coverage declined from ~4.23% to ~1.29% (Fig. 3K and L). These results establish glycan cross-linking as a biophysical mechanism driving macromolecular assembly of functional transport platforms that enable SerT/GluT co-organization.

All of the above results collectively demonstrate that lipid rafts and glycan cross-linking cooperatively stabilize SerT/GluT clusters on the plasma membrane. The assembly patterns and spatial colocalization of these transporters show strong coupling with serine homeostasis maintenance via balanced intracellular synthesis and extracellular transport.

### Metabolic reprogramming under glucose deprivation involves spatial reorganization of SerTs and GluTs

**Fig. 4.**
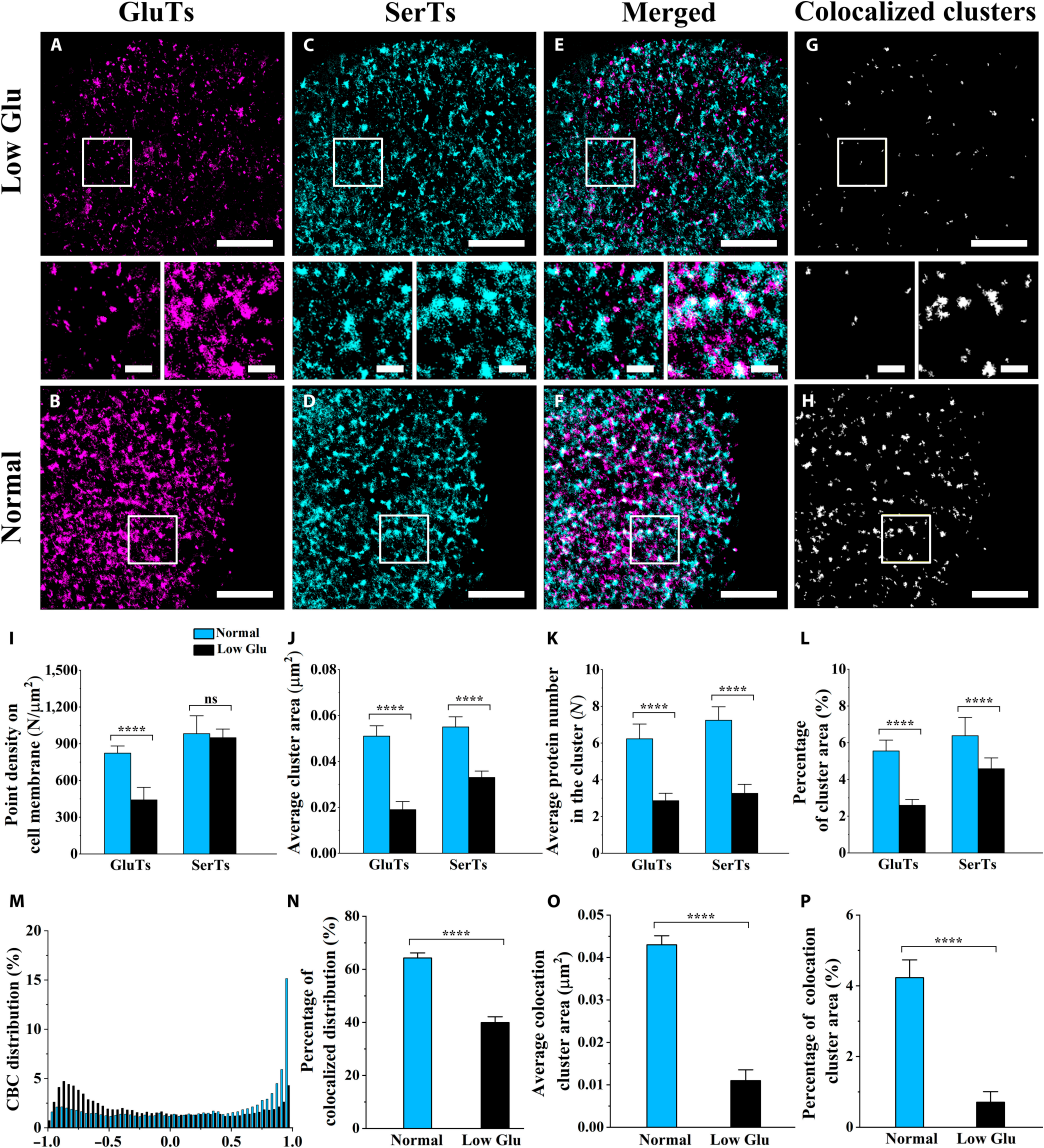
The contrast dual-color dSTORM imaging of GluT and SerT distribution on MCF7 cell membranes after low-glucose treatment. (A to D) Single-channel dSTORM reconstructions and merged images (E and F) of GluT (magenta) and SerT (cyan) distributions on normal and glucose-restricted MCF7 cells, as well as the corresponding maps of the colocalized regions (G and H). (I to L) Compared histograms of the average point density on the cell membrane (I), average cluster area (J), average protein number in clusters (K), and average cluster coverage (L) on normal and glucose-restricted MCF7 cells. (M to P) Comparison of CBC value distributions (M), the proportion of *V*_CBC_ (0 < *V*_CBC_ ≤ 1) (N), average cluster area (O), and coverage percentage (P) of the colocalized regions from normal and glucose-restricted MCF7 cells. All data are the statistical results of more than 10 cells from 3 independent experiments. Significant difference analyses were performed by using the unpaired 2-tailed *t* test, with “ns” meaning no significant difference and “****” meaning *P* < 0.0001. Scale bars are 5 μm in (A) to (H) and are 500 nm in enlarged images.

To experimentally validate the hypothesized spatial coordination between GluTs and SerTs in serine acquisition pathways, we designed a metabolic perturbation assay targeting their predicted functional linkage. Considering that glucose serves as the primary carbon source for intracellular serine biosynthesis, we subjected cells to acute glucose deprivation under a low-glucose medium (5.5 mM) to create a serine biosynthetic bottleneck [35]. Compared dSTORM imaging revealed significant dispersion of GluT clustering distribution on treated cells (Fig. 4A) compared to that on cells cultured in normal conditions (glucose 25 mM) (Fig. 4B). Quantitative analysis demonstrated a 46.42% reduction in point density on the cell membrane, a 62.75% decrease in cluster area, a 54.09% shrinkage in average protein number in clusters, and a 53.33% decrement in cluster coverage (Fig. 4I to L). In contrast, dSTORM imaging of SerTs displayed that slighter distribution changes happened with glucose shortage (Fig. 4C), compared with normal cell imaging (Fig. 4D). No statistically significant change in point density on the cell membrane (from ~982 to ~949 N/μm^2^) suggested that SerTs maintained a stable membrane distribution amount (Fig. 4I). Moreover, SerTs exhibited changes in clustered organization (Fig. 4J to L), with a decrease of 40% in cluster area, 54.97% in average protein number in clusters, and 28.21% in cluster coverage. These data indicate that glucose deprivation triggers a coordinated adaptive response involving transporter reorganization under metabolic rewiring. Glucose shortage attenuated the clustering distribution of GluTs, correlating with reduced transport efficiency. This adaptive response likely coincided with diminished glucose uptake under low-glucose conditions, potentially representing an energy-saving mechanism during nutrient scarcity [23]. Concomitantly, SerTs also exhibited reduced aggregation and increased dispersion. We speculate that this redistribution of SerTs is likely correlated with the dissolution of GluTs, due to their original substantial colocalization [36].

To further examine the correlation alterations in the spatial relationship between GluTs and SerTs in metabolic rewiring under glucose limitation, we observed the detailed spatial relationship of GluTs and SerTs on the treated cells from the merged images in dual-color dSTORM imaging. The degree of colocalization between SerTs and GluTs greatly decreased (Fig. 4E and F), which is more clearly shown in the corresponding maps of the extracted colocalized regions (Fig. 4G and H).

After CBC analysis, the proportion of the mutual correlation distribution (0 < *V*_CBC_ ≤ 1) was found to decrease from 64.27% to 39.93% (Fig. 4M and N). Through cluster analysis of the colocalized regions, compared with those of untreated cells, the average cluster area reduced from ~0.043 to ~0.011 μm^2^ and the average coverage percentage decreased from ~4.23% to ~0.71% (Fig. 4O and P). These results suggest that the degree of spatial colocalization distribution between SerTs and GluTs indeed diminishes in parallel with their declining functional correlation in maintaining the intracellular serine level through respectively regulating serine synthesis and transport under glucose starvation. That is, the spatial correlation of GluTs and SerTs is dynamically correlated with the balance between endogenous serine synthesis and exogenous uptake, highlighting the spatial topological plasticity of transporter proteins in metabolic adaptability. These findings reveal a close link between nutrient transporter organization and metabolic pathway regulation, providing new insights into cellular adaptation to energy stress.

### Membrane reorganization of targeted transporters is closely related to the efficacy effect of PHGDH inhibition

**Fig. 5.**
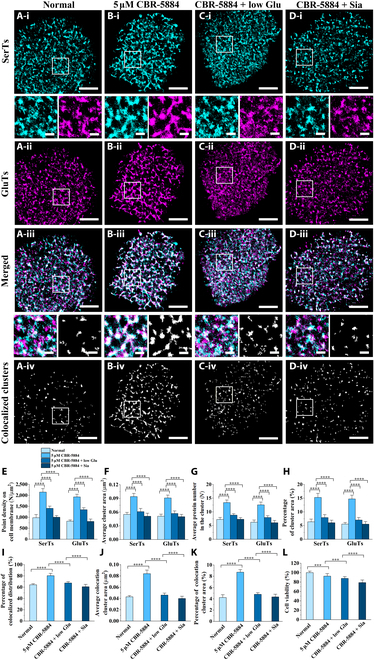
Compared dual-color dSTORM imaging of SerTs and GluTs on normal and treated cells with the inhibitor alone or combination action. (A to D) Single-channel dSTORM reconstruction images (*i* and *ii*) and merged images (*iii*) of SerT (cyan) and GluT (magenta) distributions on normal (A) and treated MCF7 cells with CBR-5884 (B) or CBR-5884 + low glucose (C) or CBR-5884 + Sia (D), as well as the corresponding maps of the colocalized regions (*iv*). (E to H) Compared histograms of the average point density on the cell membrane (E), average cluster area (F), average protein number in clusters (G), and average cluster coverage (H) on normal and treated MCF7 cells with CBR-5884 or CBR-5884 + low glucose or CBR-5884 + Sia. (I to L) Compared histograms of the proportion of *V*_CBC_ (0 < *V*_CBC_ ≤ 1) (I), average cluster area (J), and coverage percentage (K) of the colocalized regions, and the cell survival rate from Cell Counting Kit-8 (CCK-8) assay (L). All data are the statistical results of more than 10 cells from 3 independent experiments. Significant difference analyses were performed by using the unpaired 2-tailed *t* test, with “***” meaning *P* < 0.001 and “****” meaning *P* < 0.0001. Scale bars are 5 μm in original images and are 500 nm in enlarged images.

The serine biosynthesis pathway can serve as a key cancer vulnerability, with PHGDH inhibitors like CBR-5884 showing therapeutic potential [37]. However, cancer cells rapidly adapt through serine transport remodeling and compensatory anabolic activation, leading to transient proliferation bursts that limit treatment efficacy [38–40]. Our research findings establish a key link between the spatial organization of SerTs/GluTs and the balance of synthesis and uptake. This prompts us to explore whether changing their membrane assembly can affect the intracellular serine level, potentially overcoming the limitations of current serine-targeted therapies. Using CBR-5884 (a selective PHGDH inhibitor) as a representative drug targeting the serine synthesis pathway, we first investigated the correspondence between spatial assembly changes of SerTs/GluTs and drug efficacy. Single-channel dSTORM images revealed that 5 μM inhibitor treatment significantly enhanced the membrane clustering of both SerTs and GluTs compared to that in untreated cells (Fig. 5A (*i*) and (*ii*) and B (*i*) and (*ii*)), with this effect exhibiting dose-dependent attenuation (Figs. S6A to D and S7A to D). Quantitative analysis further demonstrated, with increasing drug concentration from 5 to 10 to 30 μM, progressive increases in protein distribution amount, with the point density on the cell membrane expanding by 134.39%/75.33%/22.6% for GluTs and 118.94%/61.81%/13.75% for SerTs (Figs. S6H and S7H); an enhanced protein aggregation degree was also demonstrated in cluster size and cluster compactness, with the cluster area increasing by 78.43%/43.14%/9.8% for GluTs and by 70.91%/38.18%/5.45% for SerTs (Figs. S6I and S7I); the intracluster protein count rose by 101.44%/67.42%/24.88% for GluTs and by 84.94%/49.72%/10.08% for SerTs (Figs. S6J and S7J). The overall distribution of protein clusters on the cell membrane was also becoming increasingly significant, with cluster coverage increasing by 165.23%/92.97%/29.73% for GluTs and 139.34%/77.74%/15.05% for SerTs (Figs. S6K and S7K). These changes in the distribution of SerTs and GluTs indicate initial drug-induced coalescence of transporter microdomains followed by concentration-dependent dispersion. These findings establish a dose-responsive relationship between pharmacological inhibition and transporter spatial reorganization on the plasma membrane. We propose that PHGDH inhibition triggers compensatory mechanisms to maintain serine homeostasis: (a) increased GluT clustering assembly may enhance glucose uptake to improve substrate concentration, thereby attenuating the blockade of CBR-5884 on PHGDH inhibition on endogenous synthesis, and (b) amplified SerT aggregation distribution can boost serine transport, compensating for impaired Ser synthesis. This dual response explains the observed transporter co-clustering at low inhibitor concentrations. However, at elevated drug concentrations, cellular stress responses attenuated, while cytotoxic effects intensified. Consequently, the drug-induced enhancement of transporter clustering assembly observed at lower concentrations diminished. This loss of adaptive clustering correlates with reduced cellular nutrient demand during active damage (as quantified by Cell Counting Kit-8 [CCK-8] assays in Fig. S8K), resulting in attenuated clustering assembly of both transporters concomitant with diminished transport capacity. Collectively, these data indicate that the stimulatory effect of high-concentration drug exposure on protein clustering progressively weakens. This dual-phase adaptation pattern, where compensatory transporter clustering precedes dose-responsive inhibition, reveals the spatial reorganization of transporters under metabolic stress.

The merged images in dual-color dSTORM imaging revealed that inhibitor treatment significantly enhanced the colocalization of SerTs and GluTs compared to that in untreated cells, as evidenced in both merged images and magnified views (Fig. 5A (*iii*) and B (*iii*)). Intriguingly, the colocalization increase exhibited a dose-dependent effect, progressively attenuating at higher inhibitor concentrations (Fig. S8A (*i*) to D (*i*)). This trend was further corroborated by CBC analysis, showing *V*_CBC_ proportions of 80.44%, 74.11%, and 66.13% for ascending doses, compared with 64.27% in the untreated cells (Fig. S8H). The colocalized regions were further extracted from the corresponding merged images to clearly display the differences in overlap distribution of SerTs and GluTs as inhibitor action under increasing concentrations (Fig. S8A (*ii*) to D (*ii*)). After cluster analysis, as the inhibitor concentration increased from 5 to 10 to 30 μM, the average cluster area changed from 0.084 to 0.057 to 0.044 μm^2^, and the average cluster coverage altered from 8.72% to 6.45% to 4.24% (Fig. S8I and J). These data again show that as the concentration of the drug action increases, the proportion of colocalization of the 2 transporters decreases. Collectively, our results demonstrate a strong correlation between inhibition efficacy, individual transporter aggregation patterns, and their colocalization frequency. Upon serine synthesis blockade, both transporters exhibited rapid adaptive responses through dynamic reorganization of their spatial distributions and intertransporter positioning, suggesting metabolic-state-dependent regulation of their membrane assembly.

Given the observed inverse relationship between transporter aggregation/colocalization and drug efficacy, we hypothesized that reversing drug-induced assembly remodeling could enhance therapeutic outcomes. Building on our discovery that glucose restriction could weaken transporter clustering/colocalization, we investigated whether metabolic modulation by glucose limitation could synergize with CBR-5884 to potentiate its effects. Compared single-channel imaging demonstrated that although the dual intervention (5 μM CBR-5884 + low glucose) enhanced SerT and GluT clustering reorganization (Fig. 5C (*i*) and (*ii*)) compared to untreated cells (Fig. 5A (*i*) and (*ii*) but with a slighter increase extent than single 5 μM CBR-5884 treatment (Fig. 5B (*i*) and (*ii*)). This dual intervention increased the average membrane point density by 63.67% (GluTs) and 43.48% (SerTs), raised the cluster area by 11.76% (GluTs) and 10.91% (SerTs), improved intracluster protein numbers by 27.61% (GluTs) and 21.69% (SerTs), and incremented cluster coverage by 26.67% (GluTs) and 24.14% (SerTs) (Fig. 5E to H). The merged images of SerTs and GluTs display that the combination action leads to a slight enhancement in the colocalization distribution of SerTs and GluTs (Fig. 5I), with *V*_CBC_ increasing only from 64.27% (normal cell) to 67.45% (treated cells). The distribution of corresponding colocalized regions more clearly showed the changes in their colocalized spatial relationship (Fig. 5A (*iii*) and C (*iii*)), with increases only of 6.98% in average cluster area (Fig. 5J) and of 13.48% in average cluster coverage (Fig. 5K). Meanwhile, the corresponding CCK-8 results show that cellular activities diminished to 87.71% in cells treated by combined actions (Fig. 5L). These results demonstrate that glucose restriction can synergistically enhance CBR-5884’s pharmacological effects by counteracting drug-induced transporter clustering dynamics.

We next examined the dose-dependent effects of CBR-5884 combined with glucose limitation on transporter reorganization (Figs. S6E to G and S7E to G). As CBR-5884 concentrations increased (5 → 10 → 30 μM), we observed progressively attenuated drug-induced transporter clustering. Statistical analysis revealed dose-dependent distribution changes in average membrane point density (GluTs: 1,347 → 864 → 658 N/μm^2^; SerTs: 1,409 → 992 → 712 N/μm^2^), cluster area (GluTs: 0.057 → 0.049 → 0.023 μm^2^; SerTs: 0.061 → 0.053 → 0.023 μm^2^), intracluster protein count (GluTs: 7.95 → 6.72 → 4.28 N; SerTs: 8.81 → 7.17 → 4.47 N), and membrane coverage (GluTs: 7.03% → 5.87% → 3.78%; SerTs: 7.92% → 6.32% → 3.84%) (Figs. S6H to K and S7H to K). The corresponding merged images also displayed concentration-dependent dissociation of SerT–GluT complexes under glucose limitation (Fig. S8E (*i*) to G (*i*)), with 0 < *V*_CBC_ ≤ 1 decreasing from 67.45% to 44.86% through 61.92% (Fig. S8H) and the colocalized cluster area shrinking from 0.046 to 0.02 through 0.039 μm^2^ (Fig. S8I), as well as cluster coverage diminishing from 4.8% to 1.37% through 4.04% (Fig. S8J). The changes in their colocalized spatial relationship were more clearly revealed by the distribution of corresponding colocalized regions (Fig. S8E (*ii*) to G (*ii*)). These results show that the degree of increase in the aggregation distribution of SerTs and GluTs and their colocalization is becoming smaller and smaller, and even some analysis parameters have shown negative growth, indicating complete reversal of drug-induced clustering at higher CBR-5884 concentrations. This assembly remodeling correlated with enhanced cytotoxicity, as CCK-8 assays demonstrated significantly lower viability under combination therapy (87.71% → 82.65% → 76.19%) versus monotherapy (92.55% → 89.69% → 83.41%) (Fig. S8K). The results show that combining glucose restriction with PHGDH inhibition significantly disrupts the clustering assembly of SerTs and GluTs, suggesting a synergistic metabolic vulnerability. The enhanced disruption under combined treatment indicates that glucose restriction sensitizes cells to PHGDH inhibition, potentially by limiting de novo synthesis of serine, supporting a promising strategy for targeting cancer cell metabolism.

Given the assembly–function relationship between transporter clustering and drug efficacy, we investigated whether directly modulating their spatial organization/colocalization could enhance therapeutic outcomes. Since both transporters are glycosylated and their clusters can be stabilized by glycan cross-linking [38–40], we selected Sia as an exogenous monosaccharide to examine whether it can serve as a competitive binding molecule to disrupt interglycoprotein cross-linking. Firstly, the nontoxicity of free Sia treatment was confirmed by CCK-8 assays, showing 97.1% cell viability (Fig. S9L). Then, dual-color dSTORM super-resolution imaging revealed that Sia treatment substantially reduced both individual and colocalized clustering of SerTs and GluTs on plasma membranes (Fig. S9A to D), with quantitative analysis demonstrating concurrent reductions in membrane point density (SerTs: 47.35%; GluTs: 44.71%), cluster area (SerTs: 52.73%; GluTs: 56.86%), intracluster protein counts (SerTs: 35.22%; GluTs: 27.61%), and membrane coverage (SerTs: 36.83%; GluTs: 35.32%) (Fig. S9E to H). Concomitantly, colocalization analysis revealed diminished spatial correlation (*V*_CBC_ 0 to 1) from ~64.27% to 50.74%, decreased mean colocalized cluster area (~0.043 to ~0.021 μm^2^), and reduced colocalized membrane coverage (~4.23% to ~2.08%) (Fig. S9I to K), collectively indicating that Sia disrupts glycan-stabilized transporter microdomains, driving disassembly of both individual and colocalized SerT–GluT complexes.

Based on these findings, we further combined free Sia with CBR-5884 to examine the therapeutic efficacy. Super-resolution dSTORM imaging then demonstrated that cotreatment (CBR-5884 + Sia) indeed significantly attenuated SerTs (Fig. 5D (*i*)) and GluTs (Fig. 5D (*ii*)) compared to CBR-5884 alone (Fig. 5B (*i*) and (*ii*)). Quantitative analysis of membrane distribution patterns showed consistent reductions across all parameters: average membrane point density decreased by 53.3% (SerTs) and 57.96% (GluTs); cluster area diminished by 45.74% and 45.05%; intracluster protein numbers declined by 45.41% and 51.87%; and membrane coverage decreased by 60.45% and 62.5%, respectively (Fig. 5E to H). Additionally, the merged images further displayed that CBR-5884 + Sia treatment (Fig. 5D (*iii*) and (*iv*)) significantly reduced the co-clustering of SerTs and GluTs compared to CBR-5884 alone (Fig. 5B (*iii*) and (*iv*)), with the 0 < *V*_CBC_ ≤ 1 coefficient decreasing from 80.44% (monotherapy) to 60.87% (combination) (Fig. 5I), and smaller colocalized domains with average cluster area decreasing by only 52.38% and coverage by 50.23%—markedly less than with CBR-5884 alone (Fig. 5J and K). Consistent with enhanced drug efficacy, CCK-8 assays showed cell viability declining to 78.84% from combination therapy compared with 92.55% from monotherapy (Fig. 5L).

Our comparative analyses demonstrate that cotreatment with Sia and the inhibitor yields superior therapeutic outcomes compared to those of inhibitor monotherapy. This synergy again suggests that membrane transporters (SerTs and GluTs) are regulated by carbohydrate-mediated cross-linking, which promotes the formation of large protein clusters to sustain high substrate uptake. The combined Sia–inhibitor treatment appears to enhance therapeutic efficacy through targeted modulation of transporter oligomerization. This work establishes a “glycan competition-declustering” strategy that amplifies metabolic inhibition through nontoxic modulation of transporter spatial organization, simultaneously creating quantifiable membrane biomarkers.

## Conclusion

Reprogrammed serine metabolism plays a pivotal role in tumorigenesis, making the key molecules in its metabolic pathways attractive targets for cancer detection and therapy. While pharmacological inhibition of serine synthesis enzymes (e.g., PHGDH) shows therapeutic potential, critical questions remain unresolved, including the systemic role of transmembrane transport in sustaining oncogenic serine flux, and the functional interplay between de novo synthesis and solute-carrier-mediated transport in governing serine pool homeostasis under metabolic stress. In our work, we successfully synthesized a substrate-based fluorescent probe (Ser-probe) to specifically label SerTs. dSTORM imaging and quantitative flow cytometry reveal that malignant transformation drives reorganization of SerTs into larger and densely packed clusters, correlating with enhanced serine uptake efficiency. This clustering phenotype is further amplified in serine-rich environments, where intensified SerT aggregation coincides with improved substrate absorption. These findings establish aggregated SerTs as tunable functional platforms that optimize transport efficiency in cells. To mechanistically understand the differential SerT distribution patterns between cell types, we investigated how intracellular serine biosynthesis capacity modulates SerT surface organization. Using dual-color dSTORM with Ser-probe and Glu-probe labeling, we found that MCF7 cells with higher endogenous serine synthesis showed more significant GluT clustering and colocalization with SerTs, indicating that the spatial organization and colocalization patterns of SerTs and GluTs directly correlate with cellular reliance on serine synthesis versus transport, reflecting their relative contributions to serine pool maintenance. SerT and GluT clustering assembly and colocalization on the plasma membrane were revealed to mechanistically correlate with lipid raft microdomains and glycan cross-linking networks. We propose that these proteins coalesce through lipid–glycan cooperativity into collaborative assembly platforms, with their spatial reorganization correlating with functional adaptations in serine metabolism. Through altering glucose availability in MCF7 cells to modulate the serine de novo synthesis, we found that glucose shortage reduced GluT clustering assembly and weakened GluT–SerT colocalization, which further confirms functional coordination between SerTs and GluTs in maintaining intracellular serine flux. Considering that the spatial arrangement of 2 transporters is closely related to serine metabolism through serine synthesis and external absorption pathways, we further investigated the effect of drugs (CBR-5884 and PHGDH inhibitor) targeting serine synthesis on the spatial distribution of the 2 transporters. Pharmacological inhibition of serine biosynthesis produced concentration-dependent reductions in both SerT/GluT clustering and their colocalization, establishing a direct link between inhibitor efficacy and transporter spatial organization. To determine if glucose restriction potentiates CBR-5884’s regulatory effects on transporter organization, we designed a dual metabolic intervention combining the PHGDH inhibitor CBR-5884 with glucose restriction. The dual targeting of serine and glucose metabolic pathways synergistically reduced transporter clustering and colocalization, with combination therapy demonstrating superior efficacy over monotherapy. Since both transporters are glycosylated and their clusters are stabilized by glycan cross-linking, we finally observed that combining Sia with CBR-5884 could reduce clustering assembly of both SerTs and GluTs and their colocalization, demonstrating that carbohydrate-mediated cross-linking is essential for transporter cluster formation and that directed modulation of this membrane organization of targeted transporters contributes to the therapeutic effect of PHGDH inhibition.

In summary, the results redefine cancer’s metabolic dependencies by demonstrating that (a) transporter spatial topology encodes metabolic plasticity, (b) serine flux regulation operates through integrated synthesis–transport circuitry, and (c) cotargeting enzymatic and topological regulators achieves synergistic tumor suppression. By decoding the architectural logic of serine metabolic networks, this study provides a blueprint for next-generation therapies that simultaneously disrupt biosynthesis and transport adaptation mechanisms.

## Materials and Methods

### Cell culture

MCF10A, MCF7, and MDA-MB-231 were cultured according to the requirements of cell culture instructions. For details, refer to the Supplementary Experiment Section in the Supplementary Materials.

### Cell uptake experiment

Proliferative MCF7 or MDA-MB-231 cells were seeded into a 6-well plate with 5 × 10^4^ cells per well. They were incubated at 37 °C in a humidified atmosphere with 5% CO_2_. Cells were then cultured in medium either with or without 20 μM FITC–serine. After 1 h of incubation, the cells were trypsinized, washed twice with phosphate-buffered saline (1,500 rpm, 5 min), and transferred to cytometry tubes. In the case of the serine addition study, the cells were pretreated with free serine (2 mM) for 24 h before FITC–serine treatment. The samples were measured on a flow cytometer (CytoFLEX) with acquisition of 5,000 events using 488- and 525-nm excitation and emission wavelength signals with the FITC channel. The data were analyzed with compatible Accuri software, and results are expressed as the change in MFI. Untreated cells served as the negative control.

### CCK-8 assay

The CCK-8 assay was performed according to the manual provided by Sigma-Aldrich (96992) with some modifications. Cell suspensions were plated onto 96-well plates (100 μl per well). The perimeter wells (rows A and H; columns 1 and 12) were spiked with phosphate-buffered saline due to edge effects (evaporation). Each well received treatment with CBR-5884 (5, 10, and 30 μM), 20 μM Sia, or low-glucose Dulbecco’s modified Eagle medium without serum in the absence or existence of 5, 10, and 30 μM CBR-5884 for 3 h, respectively, or a combination of 5 μM CBR-5884 and 20 μM Sia for 3 h. Following treatment, 10 μl of the prepared CCK-8 solution (TargetMol) was added to the wells followed by a 45-min incubation period. Optical density measurements were taken using an enzyme labeler, with the wavelength adjusted to 450 nm.

### dSTORM imaging

An imaging sequence of 8,000 frames (512 × 512 pixels, 160 nm/pixel) was acquired by using Micro-Manager based on ImageJ (US National Institutes of Health) with a high-sensitivity electron-multiplying charge-coupled device camera (Photometrics, Cascade II) with an exposure time of 20 ms. The detailed procedure is described in the Supplementary Materials.

### Data analysis

The raw data were analyzed by ThunderSTORM based on ImageJ to yield qualified localizations and reconstruct dSTORM images (the detailed parameters can be seen in the Supplementary Materials). The localization density on the cell membrane and cluster analysis by SR-Tesseler were implemented as previous articles reported [41]. The detailed procedure is described in the Supplementary Materials.

## Supplementary Material

20250508-1

## Data Availability

All data of this study are available from the corresponding authors upon request.

## Supplementary Materials

Supplementary 1 Supplementary Experiment Section Figs. S1 to S9
