# Erratum to “Mechanistic Insight into Serine Flux Regulation through Nanoscale Organization of Glucose and Serine Transporters by Substrate Probe-Based Direct Stochastic Optical Reconstruction Microscopy Imaging”

**DOI:** 10.34133/research.1037

**Published:** 2025-12-16

**Authors:** Pengwei Jiang, Hao Hou, Jiaqi Wang, Xumin Wang, Yaqi Wang, Simin Liu, Junling Chen, Hongda Wang, Feng Liang

**Affiliations:** ^1^ The State Key Laboratory of Refractories and Metallurgy, School of Chemistry & Chemical Engineering, Wuhan University of Science and Technology, Wuhan, Hubei 430081, P. R. China.; ^2^ Research Center of Biomembranomics, Key Laboratory of Electroanalytical Chemistry, Changchun Institute of Applied Chemistry, Chinese Academy of Sciences, Changchun, Jilin 130022, P. R. China.

The authors would like to correct two errors in the Research Article entitled “Mechanistic Insight into Serine Flux Regulation through Nanoscale Organization of Glucose and Serine Transporters by Substrate Probe-Based Direct Stochastic Optical Reconstruction Microscopy Imaging”. [1]

A typo was present in the affiliation for Dr. Hongda Wang. The correct affiliation is:

Research Center of Biomembranomics, Key Laboratory of Electroanalytical Chemistry, Changchun Institute of Applied Chemistry, Chinese Academy of Sciences, Changchun, Jilin 130022, P. R. China

Revisions were also needed to the funding statement in order to accurately reflect Dr. Wang’s sources of funding. The corrected statement is below:

**Funding:** We acknowledge the financial support from the National Key R&D Program of China (No. 2024YFA1307400 to H.W.), the Natural Science Foundation of Hubei Province (no. 2023AFB595 for J.C.); and National Natural Science Foundation of China (no. 22327808 to H.W., no. 21807083 to J.C.); Key-Area Research and Development Program of Guangdong Province (no. 2022B303040003 to H.W.); Provincial College Student’s Innovation and Entrepreneurship Training Program (no. S202310488107 to J.C.).

These errors have now been corrected in the HTML and PDF versions online.
