# Supplementary material for: Mechanistic Insight into Serine Flux Regulation through Nanoscale Organization of Glucose and Serine Transporters by Substrate Probe-Based Direct Stochastic Optical Reconstruction Microscopy Imaging
Source: Research (Wash D C). 2025 Aug 5;8:0805. doi: 10.34133/research.0805 (PMC12322523; doi:10.34133/research.0805)
Supplement: Supplementary 1 — Supplementary Experiment Section Figs. S1 to S9 [file research.0805.f1.docx]

**Supporting information**

**Mechanistic insight into serine flux regulation through nanoscale organization of glucose and serine transporters by substrate probe-based dSTORM imaging**

Pengwei Jiang^1†^, Hao Hou^1†^, Jiaqi Wang^1^, Xumin Wang^1^, Yaqi Wang^1^, Simin Liu^1^, Junling Chen^1*^, Hongda Wang^2*^, Feng Liang^1*^

*a*. The State Key Laboratory of Refractories and Metallurgy, School of Chemistry & Chemical Engineering, Wuhan University of Science and Technology, 947 Heping Street, Wuhan, Hubei 430081, China. E-mail: chenjunling@wust.edu.cn of J. Chen; feng_liang@whu.edu.cn of F. liang.

*b*. State Key Laboratory of Electroanalytical Chemistry, Changchun Institute of Applied Chemistry, Chinese Academy of Sciences, Research Center of Biomembranomics, 5625 Renmin Street, Changchun, Jilin 130022, China. E-mail: [hdwang@ciac.ac.cn](mailto:hdwang@ciac.ac.cn) of H. Wang

†These authors contributed to the work equally and should be regarded as co-first authors.

*These authors are co-corresponding authors.

**Table of contents**

[Fig. S1 Imaging of SerTs with different concentrations of Ser-probe S-3](#_Toc11898)

[Fig. S2 Compared distribution features of SerTs on the membranes of different cells S-3](#_Toc26247)

[Fig. S3 Changes in the distribution of SerTs on MCF7 cell membranes following serine supplementation S-4](#_Toc3859)

[Fig. S4 Fluorescence analysis of FITC-Ser uptake by flow cytometry in MCF7 and treated cells S-5](#_Toc10117)

[Fig. S5 Comparison of V_CBC_ distribution histograms between untreated MCF7 cells and MCF7 cells treated with MβCD or PNGase F S-5](#_Toc23388)

[Fig. S6 Compared dSTORM imaging of GluTs on normal and treated cells with inhibitor alone or combination with low glucose S-6](#_Toc28158)

[Fig. S7 Compared dSTORM imaging of SerTs on normal and treated cells with inhibitor alone or combination with low glucose S-7](#_Toc31379)

[Fig. S8 Compared dual-color dSTORM imaging of SerTs and GluTs on normal and treated cells with inhibitor alone or combination with low glucose S-8](#_Toc23186)

[Fig. S9 The compared dual-color dSTORM imaging of GluTs and SerTs distribution on MCF7 cells with Sia treatment or not S-9](#_Toc28617)

[Supplementary Experiment Section S-10](#_Toc32059)

[*Synthesis of Ser-probe* S-10](#_Toc26515)

[*Cell culture* S-11](#_Toc17072)

[*Treatments* S-11](#_Toc11359)

[*Sample preparation for single-color dSTORM imaging* S-11](#_Toc25075)

[*Sample preparation for dual-color dSTORM imaging* S-12](#_Toc32325)

[*dSTORM imaging* S-12](#_Toc29522)

[*Data analysis* S-12](#_Toc29327)

[*NMR spectra* S-13](#_Toc25694)

**Supporting Figures**


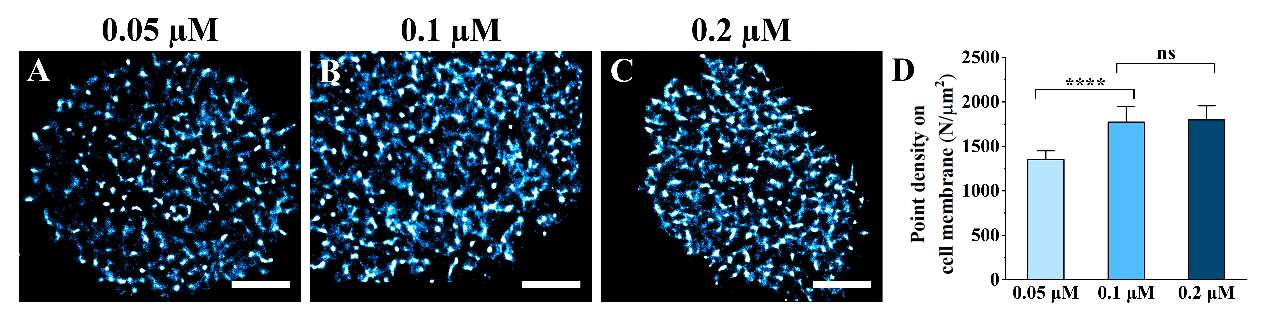


Fig. S1 Imaging of SerTs with different concentrations of Ser-probe. (A-C) A series of dSTORM reconstructed images of SerTs on the MDA-MB-231 membranes under the increasing labeling concentration of Ser-probe. Scale bars are 5 μm. (D) Compared histograms of the average point density on cell membrane under the different labeling concentrations. All data are the statistical results of more than ten cells from three independent experiments. The significant difference analyses were performed by the unpaired dual-tailed t-test, with “****” meaning P < 0.0001 and “ns” meaning no significant difference.


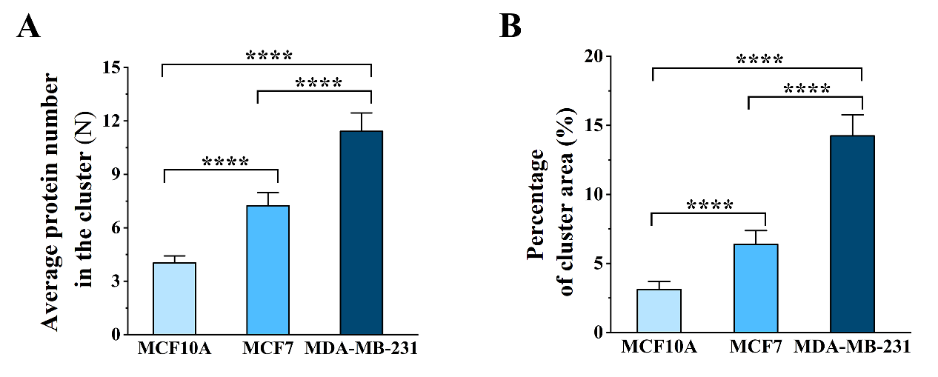


Fig. S2 Compared distribution features of SerTs on the membranes of different cells. The compared histogram of the average protein number in cluster (A) and average percentage of the cluster (B) to overall cell membrane. All data were acquired from ten cells in three independent experiments. Significant differences were analyzed by unpaired dual-tailed t-test, with “****” meaning P < 0.0001.


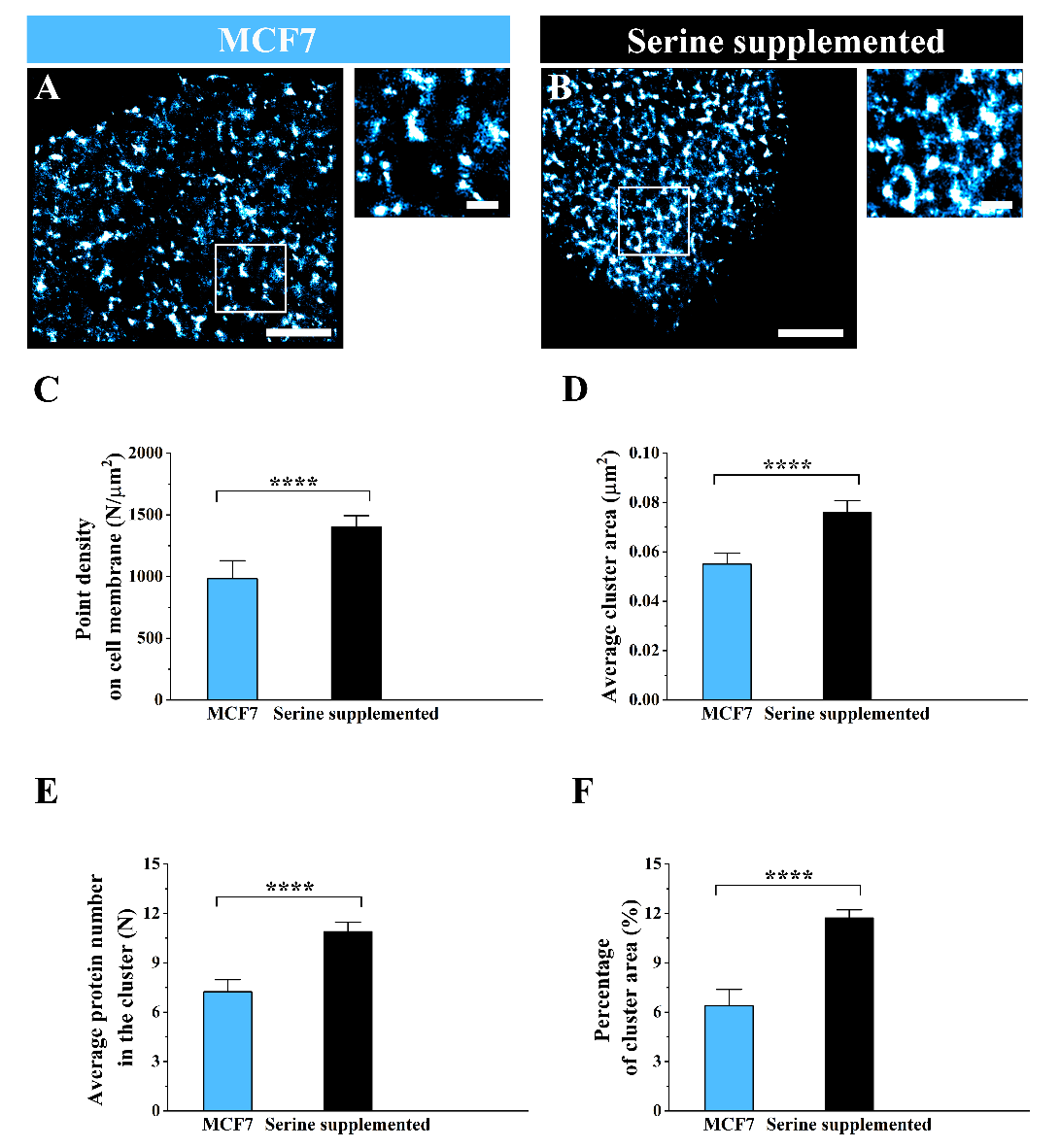


Fig. S3 Changes in the distribution of SerTs on MCF7 cell membranes following serine supplementation. (A and B) Reconstructed dSTORM images of SerTs on normal MCF7 cells (A), and on cells treated with serine supplementation (B). (C-F) Compared histograms of the average point density on cell membrane (C), average cluster area (D), average protein number in cluster (E), and average cluster coverage (F) on normal and serine-supplemented MCF7 cells. All data were obtained from ten cells in three independent experiments. The significant difference analyses were performed by the unpaired two-tailed t-test, with “****” meaning P < 0.0001. Scale bars: 5 μm in original images and 500 nm in enlarged images.


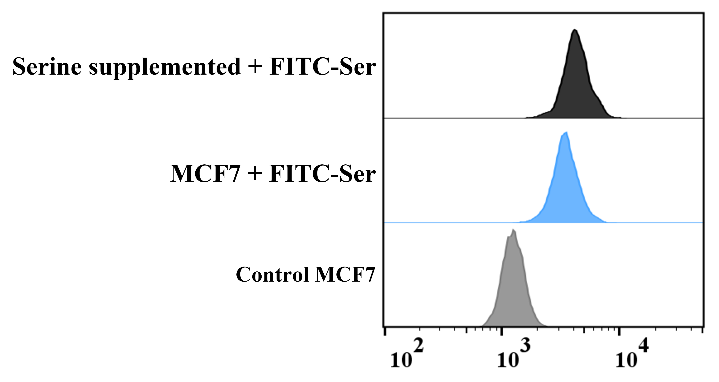


Fig. S4 Fluorescence analysis of FITC-Ser uptake by flow cytometry in MCF7 and treated cells.


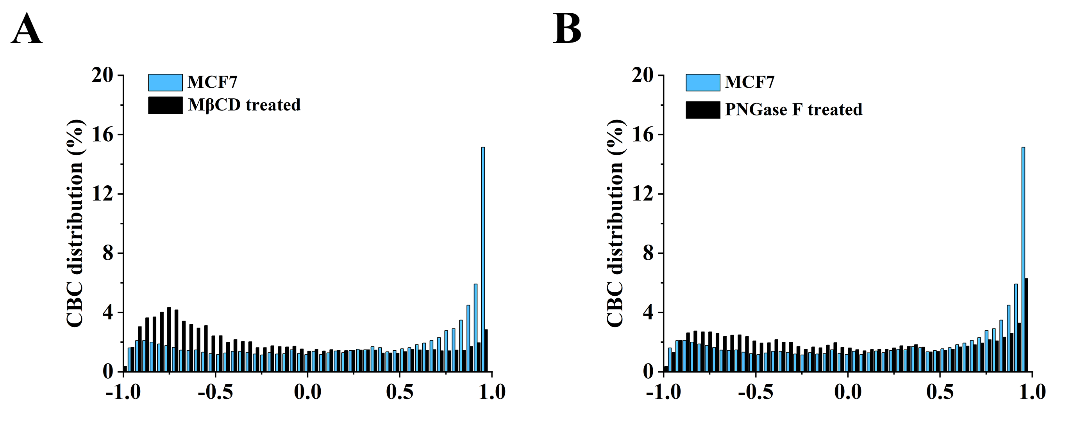


Fig. S5 Comparison of V_CBC_ distribution histograms between untreated MCF7 cells and MCF7 cells treated with MβCD (A) or PNGase F (B).


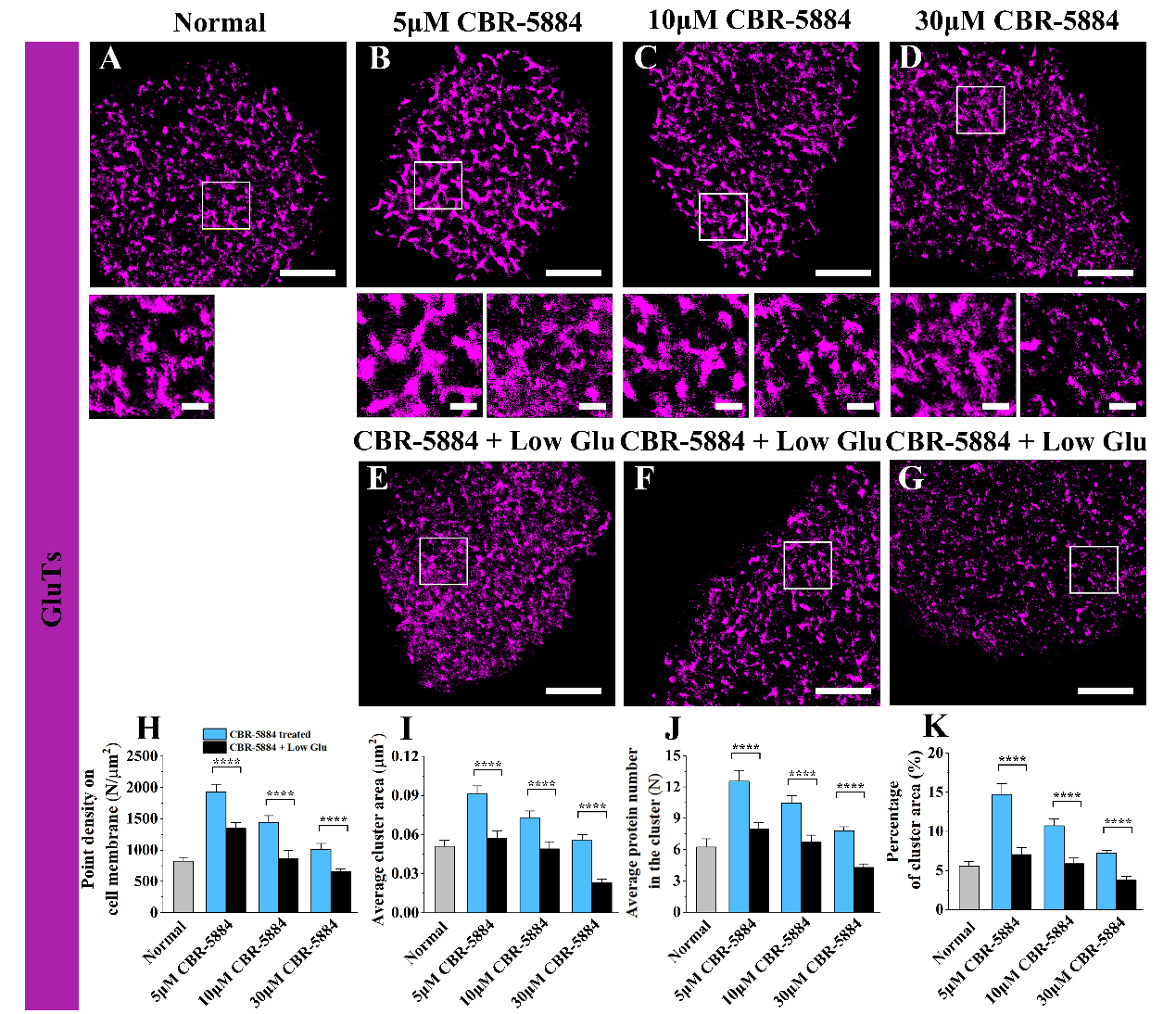


Fig. S6 Compared dSTORM imaging of GluTs on normal and treated cells with inhibitor alone or combination with low glucose. (A-D) dSTORM reconstructed images of GluTs distributions on normal (A) and treated MCF7 cells with 5 μM CBR-5884 (B), 10 μM CBR-5884 (C), 30 μM CBR-5884 (D), 5 μM CBR-5884+Low Glu (E), 10 μM CBR-5884+Low Glu (F), and 30 μM CBR-5884+Low Glu (G). (H-K) Compared histograms of the average point density on cell membrane (H), average cluster area (I), average protein number in cluster (J), average cluster coverage (K) on normal and treated MCF7 cells. All data are the statistical results of more than ten cells from three independent experiments. The significant difference analyses were performed by the unpaired two-tailed t-test, with “****” meaning P < 0.0001. Scale bars are 5 μm in original images, and are 500 nm in enlarged images.


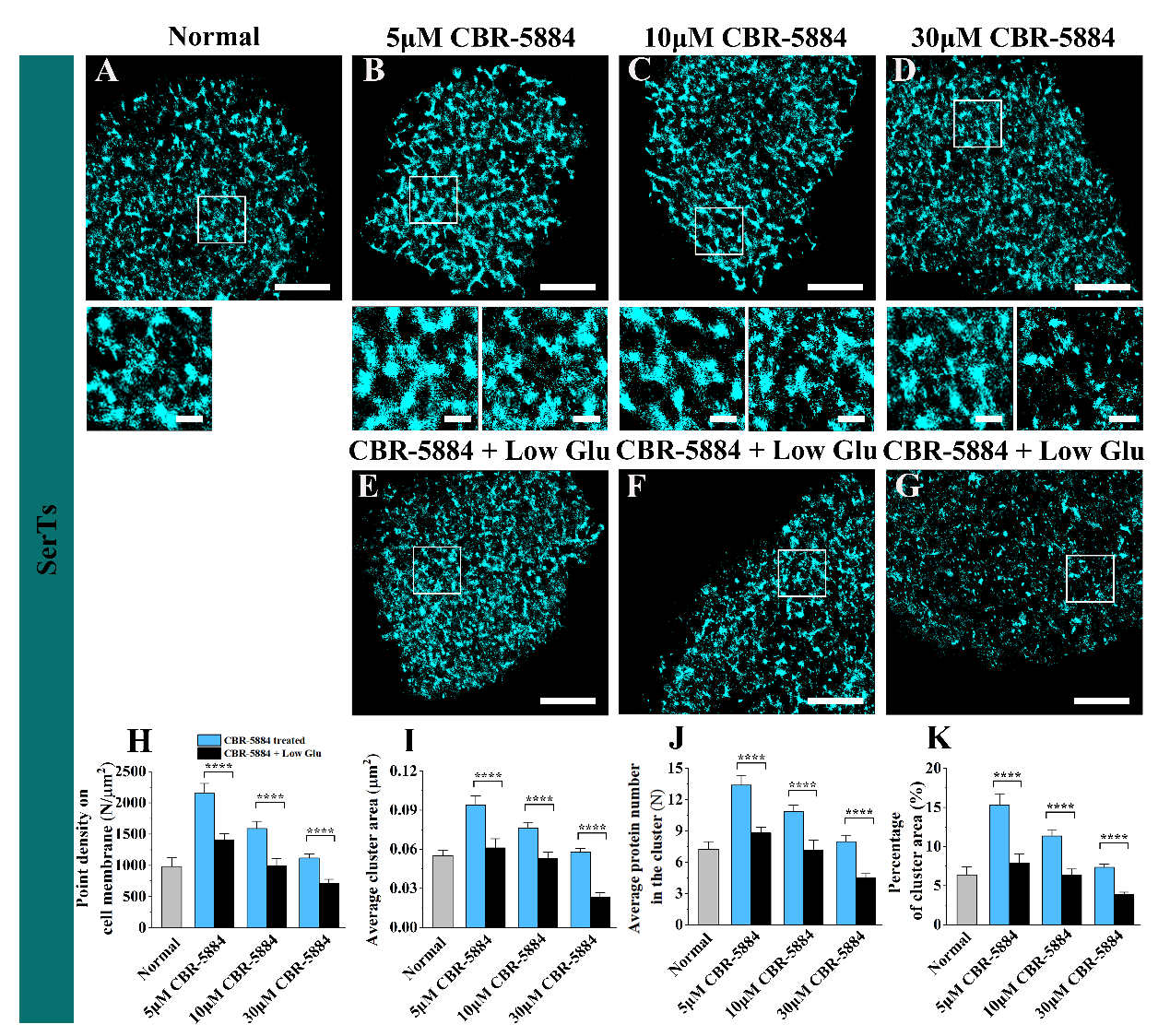


Fig. S7 Compared dSTORM imaging of SerTs on normal and treated cells with inhibitor alone or combination with low glucose. (A-D) dSTORM reconstructed images of SerTs distributions on normal (A) and treated MCF7 cells with 5 μM CBR-5884 (B), 10 μM CBR-5884 (C), 30 μM CBR-5884 (D), 5 μM CBR-5884+Low Glu (E), 10 μM CBR-5884+Low Glu (F), and 30 μM CBR-5884+Low Glu (G). (H-K) Compared histograms of the average point density on cell membrane (H), average cluster area (I), average protein number in cluster (J), average cluster coverage (K) on normal and treated MCF7 cells. All data are the statistical results of more than ten cells from three independent experiments. The significant difference analyses were performed by the unpaired two-tailed t-test, with “****” meaning P < 0.0001. Scale bars are 5 μm in original images, and are 500 nm in enlarged images.


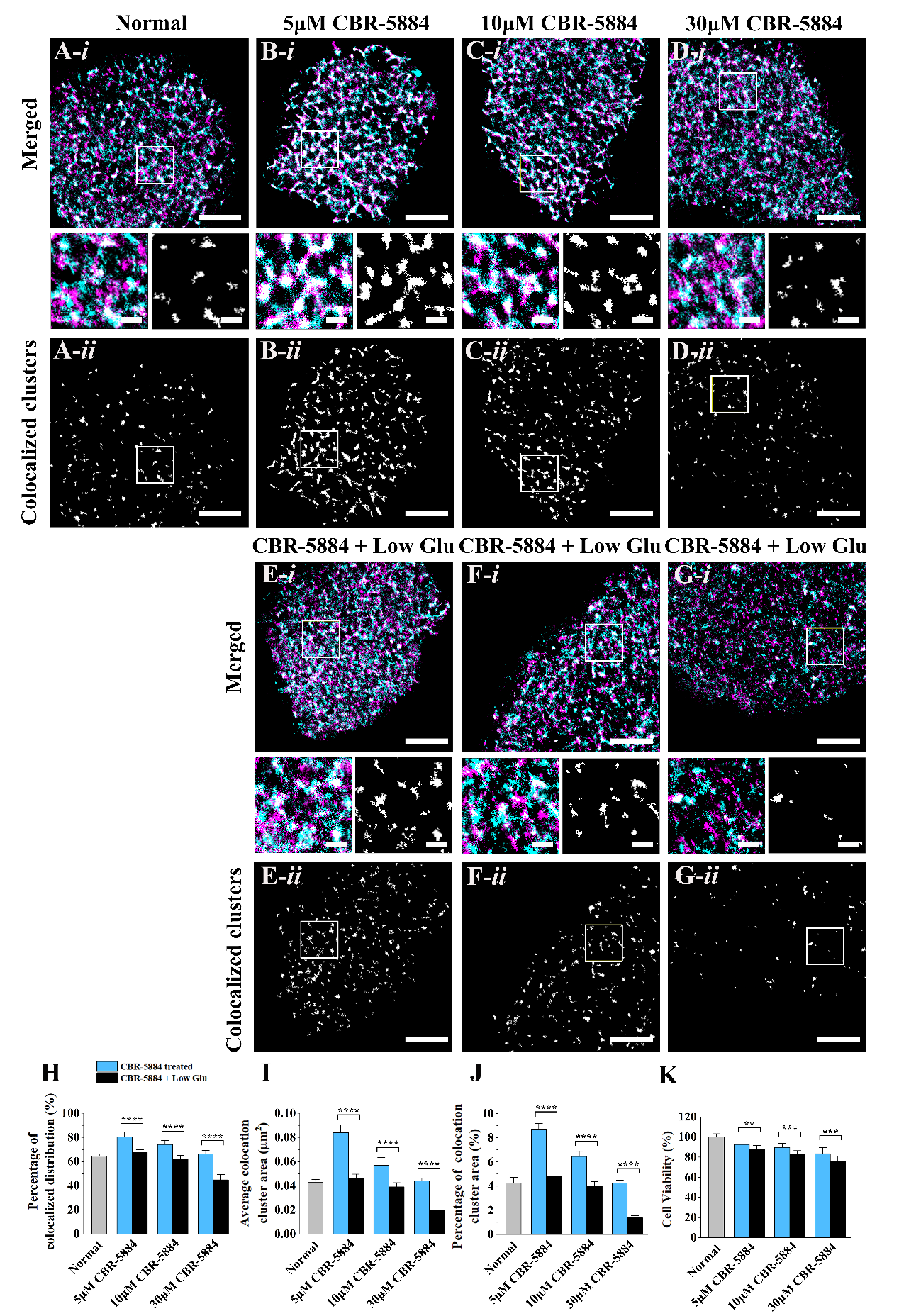


Fig. S8 Compared dual-color dSTORM imaging of SerTs and GluTs on normal and treated cells with inhibitor alone or combination with low glucose. (A-G) Merged images (*i*) of SerTs and GluTs distributions on normal (A) and treated MCF7 cells with 5 μM CBR-5884 (B), 10 μM CBR-5884 (C), 30 μM CBR-5884 (D), 5 μM CBR-5884+Low Glu (E), 10 μM CBR-5884+Low Glu (F), and 30 μM CBR-5884+Low Glu (G), as well as the corresponding maps of the colocalized regions (*ii*). (H-K) Compared histograms of the proportion of V_CBC_ (0<V_CBC_≤1) (H), average cluster area (I) and coverage percentage (J) of the co-localized regions, and the cell survival rate from CCK-8 assay (K). All data are the statistical results of more than ten cells from three independent experiments. The significant difference analyses were performed by the unpaired two-tailed t-test, with “**” meaning P < 0.01, “***” meaning P < 0.001 and “****” meaning P < 0.0001. Scale bars are 5 μm in original images, and are 500 nm in enlarged images.


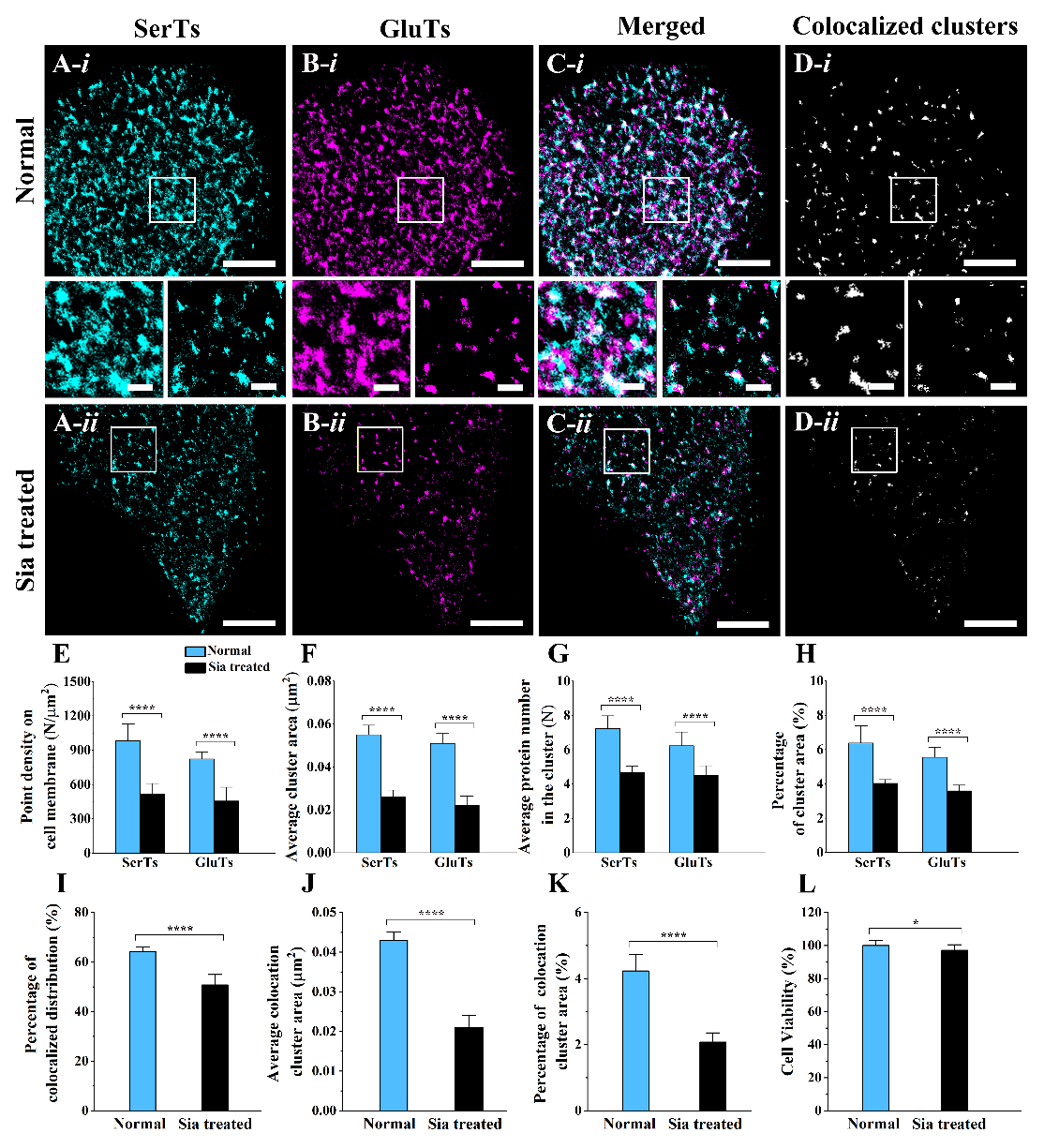


Fig. S9 The compared dual-color dSTORM imaging of GluTs and SerTs distribution on MCF7 cells with Sia treatment or not. (A and B) Single-channel dSTORM reconstructions and merged images (C) of SerTs (cyan) and GluTs (magenta) distributions on normal (*i*) and Sia-treated MCF7 cells (*ii*), as well as the corresponding maps of the colocalized regions (D). (E-H) Compared histograms of the average point density on cell membrane (E), average cluster area (F), average protein number in cluster (G), average cluster coverage (H) on normal and treated MCF7 cells with Sia. (I-L) Compared histograms of the proportion of V_CBC_ (0<V_CBC_≤1) (I), average cluster area (J) and coverage percentage (K) of the co-localized regions, and the cell survival rate from CCK-8 assay (L). All data are the statistical results of more than ten cells from three independent experiments. The significant difference analyses were performed by the unpaired two-tailed t-test, with “*” meaning P < 0.05 and “****” meaning P < 0.0001. Scale bars are 5 μm in original images, and are 500 nm in enlarged images.

**Supplementary Experiment Section**

*Synthesis of Ser-probe*

**Scheme 1: Synthesis route of Ser-probe.** Reagents and conditions: (a) PEG linker, NaH, DMF, rt, 16 h; (b) DCM/TFA (v/v = 5:1), rt, 2 h; and (c) TAMRA-Alkyne, CuSO_4_, TCEP, TBTA, PBS, rt, 12 h, dark.

General Methods: substrates and reagents are commercially available, and used as received. ^1^H and ^13^C spectra were recorded with Bruker AVX 400 MHz spectrometers in CDCl_3_ and *d_6_*-DMSO. Tetramethylsilane (*δ* = 0 ppm) was used as internal standard for ^1^H and ^13^C spectra. The structures of known compounds were confirmed by ^1^H NMR spectroscopy and comparison with literature data.

**O-(2-(2-(2-azidoethoxy)ethoxy)ethyl)-N-(tert-butoxycarbonyl)-*L*-serine (1)**

To a stirred solution of (*tert*-butoxycarbonyl)-*L*-serine (0.1 g, 0.49 mmol) and PEG linker (0.208 g, 0.73 mmol) in DMF (5 mL) at 0℃ was added NaH (0.06 g, 1.47 mmol) by portions. Then the reaction mixture was allowed to warm up to room temperature and stirred for 16 h. Cooled to 0℃, and saturated sodium carbonate was added. The mixture was extracted by DCM for three times. The organic phase was combined, washed with 1 M HCl, brine, dried over Na_2_SO_4_ and concentrated under reduced pressure. The residue was purified by column chromatography with eluent (DCM/MeOH = 10:1, v/v) to afford O-(2-(2-(2-azidoethoxy)ethoxy)ethyl)-N-(tert-butoxycarbonyl)-*L*-serine (0.08 g, 45%) as a colorless oil. ^1^H NMR (400 MHz, DMSO-*d*_6_) δ 12.65 (s, 1H), 6.82 (d, *J* = 8.1 Hz, 1H), 4.15-4.05 (m, 1H), 3.64-3.59 (m, 4H), 3.57-3.51 (m, 8H), 3.38 (d, *J* = 4.8 Hz, 2H), 1.38 (s, 9H).

**O-(2-(2-(2-azidoethoxy)ethoxy)ethyl)-*L*-serine (2)**

Compound 1 (0.044 g) was dissolved in DCM (1 mL) and TFA (0.2 mL), then the reaction mixture was stirred at room temperature for 2 h. The solvent was evaporated under reduced pressure to afford O-(2-(2-(2-azidoethoxy)ethoxy)ethyl)-*L*-serine (0.031 g) as a yellow oil. The crude product was used for next step without further purification. ^1^H NMR (400 MHz, DMSO-*d*_6_) δ 8.28 (s, 3H), 4.18 (s, 1H), 3.86 (dd, *J* = 10.8, 4.7 Hz, 1H), 3.77 (dd, *J* = 10.7, 3.2 Hz, 1H), 3.62-3.53 (m, 10H), 3.42-3.37 (m, 2H).

**Preparation of Ser-TAMRA probe**

In 100 μL of PBS, TAMRA-alkyne (4 mM) and compound 2 (2 mM) were allowed to react in the presence of CuSO_4_ (1 mM), tris(carboxyethyl)phosphine (1 mM), and ligand (2 mM). After reaction at dark for 12 hours, the reaction mixture was purified by illustra NAP-5 columns (GE Healthcare) with PBS as eluent to obtain the desired Ser-TAMRA. The combination ratio of dye to Ser was measured by UV-visible absorption spectroscopy assay. Finally, the qualified solution with a suitable ratio (~1.0) were collected for labeling experiment.

*Cell culture*

Mammary cells MCF10A were purchased from National Collection of Authenticated Cell Cultures, which were cultured with DMEM/F12 (HyClone) medium containing 5% horse serum (Gibco), epidermal growth factor (EGF) (20 ng/mL), hydrocortisone (0.5 μg/mL), cholera toxin (100 ng/mL), insulin (10 μg/mL), 100 U/mL penicillin, and 100 μg/mL streptomycin. Cells were cultured in a cell culture incubator at 37°C and 5% CO_2_. For dSTORM imaging, digested cells were cultured on clean cover slips (22 mm × 22 mm, Fisher) in dishes for at least 24 hours to achieve ~50-60% confluence.

The breast cancer cells MDA-MB-231 and MCF7 were purchased from National Collection of Authenticated Cell Cultures, which were cultured with RPMI-1640 and DMEM medium, respectively, containing 10% fetal bovine serum (FBS, HyClone), 100 U/mL penicillin, and 100 μg/mL streptomycin. Cells were cultured in a cell culture incubator at 37°C and 5% CO_2_. For dSTORM imaging, digested cells were cultured on clean cover slips (22 mm × 22 mm, Fisher) in dishes for at least 24 hours to achieve ~50-60% confluence.

*Reagent Treatments*

MβCD or PNGase F treatment: The cells were washed with prewarmed PBS one time and then were treated with methyl-β-cyclodextrin (MβCD; Sigma; 10 mM) for 30min at 37°C or with peptide-N-glycosidase F (PNGase F Sigma; 5 U/mL) for 30 min at 37°C.

Low glucose treatment: The regular culture medium was removed from the dishes with the cells. The cells were washed three times with Phosphate buffer saline (PBS) at room temperature, the low glucose DMEM medium without serum was added and the cells were incubated for 3 h at 37°C in an incubator.

CBR-5884 or Sia treatment: The cells with suitable confluence were washed three times with PBS at room temperature. The complete medium containing CBR-5884 (5, 10 and 30 μM) or 20 μM Sia was added and the cells were incubated for 3 h at 37°C in an incubator.

Drug combination: The cells with suitable confluence were washed three times with PBS at room temperature. The cells cultured in low glucose DMEM medium without serum in the presence of 5, 10 and 30µM CBR-5884, or 20µM Sia for 3h at 37°C in an incubator respectively, or a combination of 5μM CBR-5884 and 20μM Sia in complete medium for 3h at 37°C.

*Sample preparation for single-color dSTORM imaging*

For fixed cell imaging, cultured cells were fixed with 4% PFA at room temperature for 20 min. After fixation, cells were washed with PBS, blocked with 0.3% BSA at room temperature for 20 min, and then stained with Ser-probe (0.1 μM) at room temperature for 10 min, protected from light. After the stain solution removed, the cells were washed 3-4 times with PBS. 50 μL of imaging buffer was added dropwise on a slide (24 mm × 50 mm); the imaging buffer contained: Tris (50 mM, pH 8.0), NaCl (10 mM), glucose (10% w/v), glucose oxidase (500 μg/mL, sigma), catalase (40 μg/mL, sigma), β-mercaptoethanol (βME 1% v/v, sigma). Coverslips (22 mm × 22 mm) with cells attached were slowly placed on slides and sealed with nail polish.

*Sample preparation for dual-color dSTORM imaging*

After fixing and blocking as above. the sample was first stained with Glu-probe for 10 min at room temperature, then washed with PBS on a shaker for 3-4 times, and then stained with Ser-probe for 10 min. The operation to follow is the same as above.

*dSTORM imaging*

We used an inverted Nikon Ti-E microscope with an oil-immersion objective (100X, 1.49 NA, Nikon, Japan) for dSTORM imaging. Under the total internal reflection fluorescence (TIRF) illuminating mode, the sample was imaged by adjusting the excitation inclination to maximize the signal-to-noise ratio. For single color dSTORM imaging, the sample was excited with a 532 nm laser. For dual-color dSTORM imaging, the sample was firstly imaged by 639 nm laser illumination, then by 532 nm laser excitation. To avoid the color-crosstalk, two band pass emission filters (FF01-565/133-25, FF02-675/67-25, Semrock) were further added, respectively, in addition to the conventional excitation filter, dichroic mirror and emission filter set. Meanwhile, to correct the x-y drift and the optical registration between the TAMRA and the Alexa647 channels, micro-spheres (Invitrogen) were added as fiducials. Nikon micro imaging equip-ment also provides a perfect focus system (PFS) to realize the real-time correction of the focus drift in the y axis. Finally, by combining with Micro-Manager based on ImageJ (U.S. National Institutes of Health), 8000 raw frames were acquired with an EMCCD camera (IXON-L-897, Andor) with 20 ms exposure time.

*Data analysis*

**Reconstruction of dSTORM Image.**

Raw data was analyzed by ThunderSTORM to reconstruct dSTORM image. By selecting parameter thresholds and configuring the privatecamera parameters in accordance with the circumstances of our experiment, the raw data can be analyzed. After obtaining all localizations, we further set the vital parameters thresholds, including the sigma (the range of main peak), the intensity (> 200), the offset (removal of the outliers), the uncertainty (< 40), and acquired the qualified localizations with removing the “bad localization”; then, with applying the “merge” and “drift correct”, a dSTORM image with nanoscale resolution was finally reconstructed in an average shifted histogram mode.

**Cluster Analysis by SR-Tesseler.**

We applied SR-Tesseler to analyze the spatial distribution of clusters. Based on the local points density, the SR-Tesseler allows precise and automatic quantification of protein organization. Here, the dSTORM coordinates of localizations was imported firstly, then the region of interest is selected to create the Voronoï diagram. By setting the density factor, cut distance, min area and min#locs, objects were recognized. Then, clusters can be further abstracted from the objects, with setting the density factor and min area and min#locs. Following these processes, the quantitative information on protein organization morphological parameters were yielded.

**Colocalization Analysis by CBC Method.**

CBC analysis was performed to show the co-localization of dual-color dSTORM data. In the analysis, the colocations of the two channels are imported into ThunderSTORM respectively and then the dSTORM image is reconstructed, the “CBC” was applied to calculate the col-ocalization value VCBC. As VCBC increases from −1 to 1, the spatial relationship of the locus points also became closer and closer, from anti-correlated (VCBC = −1) to perfectly colocalized distribution (VCBC = 1). Finally, the percentage of VCBC greater than 0 can be used to quantify the degree of co-localization of points from two probes labeling. The simulated clusters with random distribution was analyzed by CBC method. With DBSCAN, two sets of cluster data were yielded. After importing the localization points of the two sets of simulated data into ThunderSTORM, apply “CBC” to calculate the co-localization value VCBC.

*NMR spectra*

**
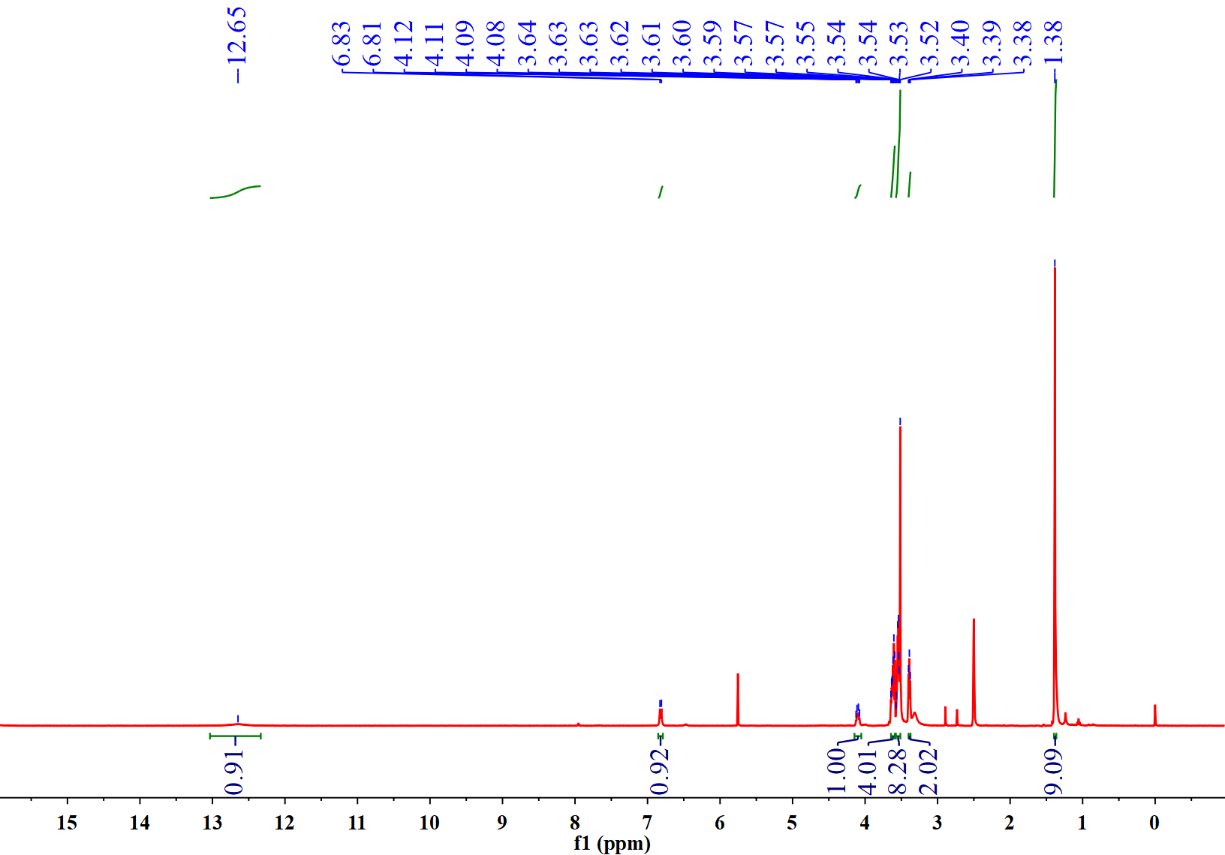
**

^1^H NMR spectra of 1 in DMSO-*d_6_* (600 MHZ, 298K).

**
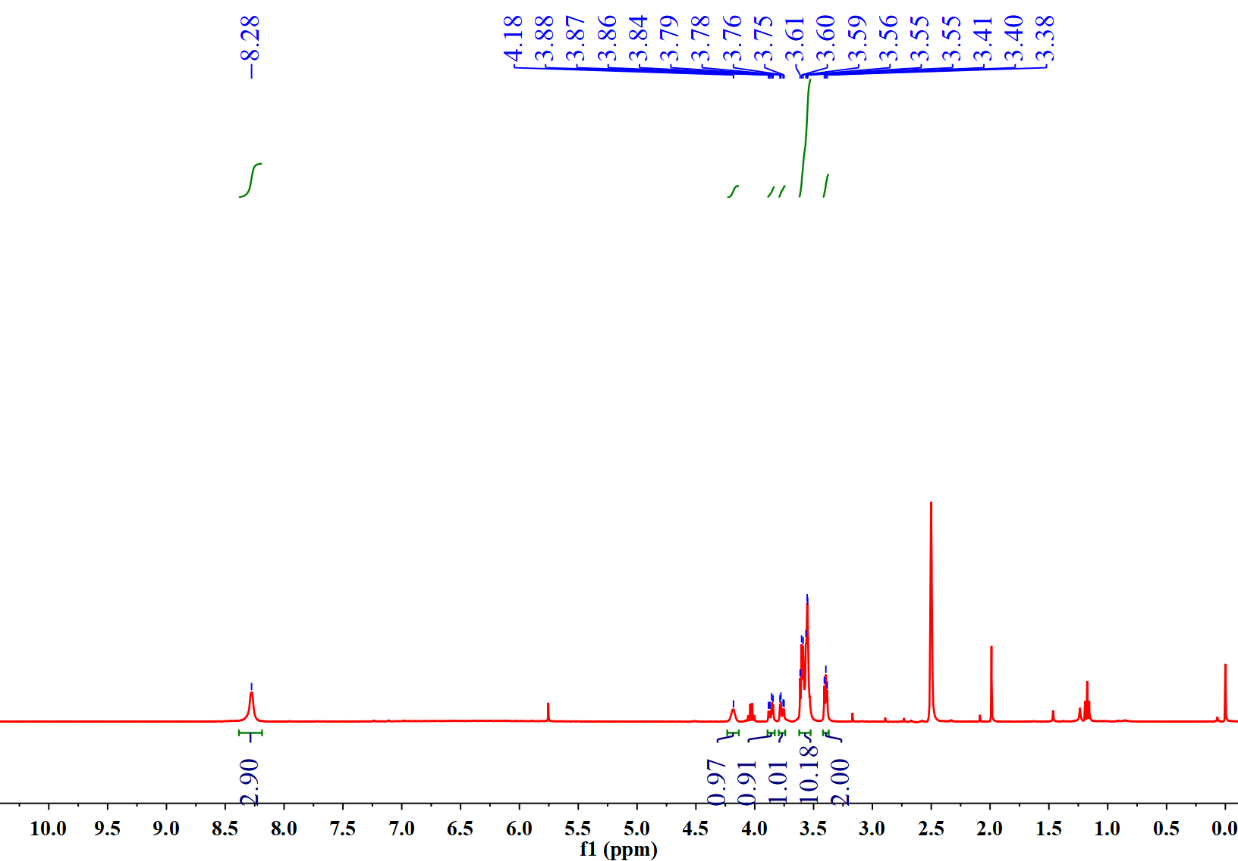
**

^1^H NMR spectra of 2 in DMSO-*d_6_* (600 MHZ, 298K).
